# Dynamics of *Salmonella enterica* and antimicrobial resistance in the Brazilian poultry industry and global impacts on public health

**DOI:** 10.1371/journal.pgen.1010174

**Published:** 2022-06-02

**Authors:** Nabil-Fareed Alikhan, Luisa Zanolli Moreno, Luis Ricardo Castellanos, Marie Anne Chattaway, Jim McLauchlin, Martin Lodge, Justin O’Grady, Roxana Zamudio, Emma Doughty, Liljana Petrovska, Marcos Paulo Vieira Cunha, Terezinha Knöbl, Andrea Micke Moreno, Alison E. Mather

**Affiliations:** 1 Quadram Institute Bioscience, Norwich, United Kingdom; 2 Department of Preventive Veterinary Medicine and Animal Health, School of Veterinary Medicine and Animal Science, University of São Paulo, São Paulo, Brazil; 3 Centro Universitário Max Planck (UniMax), Indaiatuba, São Paulo, Brazil; 4 UK Health Security Agency National Infection Service, London, United Kingdom; 5 University of East Anglia, Norwich, United Kingdom; 6 Department of Bacteriology and Food Safety, Animal and Plant Health Agency (APHA—Weybridge), Addlestone, United Kingdom; 7 Department of Pathology, School of Veterinary Medicine and Animal Science, University of São Paulo, São Paulo, Brazil; University of Warwick, UNITED KINGDOM

## Abstract

Non-typhoidal *Salmonella enterica* is a common cause of diarrhoeal disease; in humans, consumption of contaminated poultry meat is believed to be a major source. Brazil is the world’s largest exporter of chicken meat globally, and previous studies have indicated the introduction of *Salmonella* serovars through imported food products from Brazil. Here we provide an in-depth genomic characterisation and evolutionary analysis to investigate the most prevalent serovars and antimicrobial resistance (AMR) in Brazilian chickens and assess the impact to public health of products contaminated with *S*. *enterica* imported into the United Kingdom from Brazil. To do so, we examine 183 *Salmonella* genomes from chickens in Brazil and 357 genomes from humans, domestic poultry and imported Brazilian poultry products isolated in the United Kingdom. *S*. *enterica* serovars Heidelberg and Minnesota were the most prevalent serovars in Brazil and in meat products imported from Brazil into the UK. We extended our analysis to include 1,259 publicly available *Salmonella* Heidelberg and *Salmonella* Minnesota genomes for context. The Brazil genomes form clades distinct from global isolates, with temporal analysis suggesting emergence of these *Salmonella* Heidelberg and *Salmonella* Minnesota clades in the early 2000s, around the time of the 2003 introduction of the Enteritidis vaccine in Brazilian poultry. Analysis showed genomes within the *Salmonella* Heidelberg and *Salmonella* Minnesota clades shared resistance to sulphonamides, tetracyclines and beta-lactams conferred by *sul2*, *tetA* and *bla*_CMY-2_ genes, not widely observed in other co-circulating serovars despite similar selection pressures. The *sul2* and *tetA* genes were concomitantly carried on IncC plasmids, whereas *bla*_CMY-2_ was either co-located with the *sul2* and *tetA* genes on IncC plasmids or independently on IncI1 plasmids. Long-term surveillance data collected in the UK showed no increase in the incidence of *Salmonella* Heidelberg or *Salmonella* Minnesota in human cases of clinical disease in the UK following the increase of these two serovars in Brazilian poultry. In addition, almost all of the small number of UK-derived genomes which cluster with the Brazilian poultry-derived sequences could either be attributed to human cases with a recent history of foreign travel or were from imported Brazilian food products. These findings indicate that even should *Salmonella* from imported Brazilian poultry products reach UK consumers, they are very unlikely to be causing disease. No evidence of the Brazilian strains of *Salmonella* Heidelberg or *Salmonella* Minnesota were observed in UK domestic chickens. These findings suggest that introduction of the *Salmonella* Enteritidis vaccine, in addition to increasing antimicrobial use, could have resulted in replacement of salmonellae in Brazilian poultry flocks with serovars that are more drug resistant, but less associated with disease in humans in the UK. The plasmids conferring resistance to beta-lactams, sulphonamides and tetracyclines likely conferred a competitive advantage to the *Salmonella* Minnesota and *Salmonella* Heidelberg serovars in this setting of high antimicrobial use, but the apparent lack of transfer to other serovars present in the same setting suggests barriers to horizontal gene transfer that could be exploited in intervention strategies to reduce AMR. The insights obtained reinforce the importance of One Health genomic surveillance.

## Author summary

Non-typhoidal *Salmonella enterica* is a common cause of diarrhoeal disease; in humans, consumption of contaminated poultry meat is believed to be a major source. Here we determine that *S*. *enterica* serovars Heidelberg and Minnesota were the most prevalent serovars in Brazilian poultry and in poultry products imported from Brazil into the UK. However, long-term surveillance data collected by the UK Health Security Agency showed no increase in the incidence of *Salmonella* Heidelberg or *Salmonella* Minnesota in human cases of clinical disease in the UK following the increase of these two serovars in Brazilian poultry. *Salmonella* Heidelberg and *Salmonella* Minnesota clades shared resistance to sulphonamides, tetracyclines and beta-lactams conferred by *sul2*, *tetA* and *bla*_CMY-2_ genes, not widely observed in other co-circulating serovars despite similar selection pressures. The *sul2* and *tetA* genes were concomitantly carried on IncC plasmids, whereas *bla*_CMY-2_ was either co-located with the *sul2* and *tetA* genes on IncC plasmids or independently on IncI1 plasmids. These findings suggest that introduction of the *Salmonella* Enteritidis vaccine, in addition to increasing antimicrobial use, could have resulted in replacement of salmonellae in Brazilian poultry flocks with serovars that are more drug resistant, but less associated with disease in humans in the UK.

## Introduction

*Salmonella enterica* is one of the commonest agents of foodborne bacterial illness [1] with poultry being an important reservoir. In a world of globalised food production, *Salmonella* can cross borders through imported food and present a potential risk for public health. Many countries in Europe, including the United Kingdom, import a large amount of poultry from Brazil, which is the largest exporter of chicken meat globally [2,3]. Previous studies have reported the presence of *S*. *enterica* serovars in imported chicken from Brazil [4,5].

Two *Salmonella enterica* serovars—Heidelberg and Minnesota—have risen to prominence in Brazilian chicken from a formerly low prevalence [6–8] and are currently predominant *Salmonella* serovars in that setting [4,5,9–11]. These changes in serovar prevalence occurred after the introduction of a vaccine against *Salmonella enterica* serovar Enteritidis in 2003, the major *Salmonella* serovar in Brazilian poultry and exported chicken meat at that time [12–14], but the changes were also likely influenced by antimicrobial use (AMU) [15]. A similar scenario has been described in the United States where targeted efforts against *Salmonella* Enteritidis promoted the rise of other serovars, such as Heidelberg and Kentucky [16]. Thus, the changes in serovar prevalence were likely influenced by vaccine coverage as well as AMU and antimicrobial resistance (AMR).

Brazilian poultry production occurs in intensive, vertically integrated systems that are highly dependent on AMU, and the increased worldwide demand for animal protein may double the antimicrobial usage in livestock in Brazil by 2030 [17]. Antimicrobial use is one of the major drivers of AMR [18] and likely plays a role in the emergence of AMR in Brazilian poultry production. *Salmonella* Heidelberg and *Salmonella* Minnesota are frequently resistant to third generation cephalosporins, tetracyclines and/or sulphonamides [6,7,19,20]. These resistance phenotypes are commonly associated with carriage of the *bla*_CMY-2_, *tet*(A) and *sul2* genes respectively [20]. These genes are commonly carried on IncI1 or IncC, formerly known as IncA/C [21], plasmids [8,19,22].

European studies have assessed the occurrence of *Salmonella* and AMR in imported chicken meat and described AMR *Salmonella* Heidelberg and/or *Salmonella* Minnesota as the predominant *Salmonella* serovars in imported chicken meat from Brazil [4,9,10]. A 2019 report in the UK described large quantities of chicken meat contaminated with *Salmonella* being imported into the country from Brazil [23]. A comparison of *Salmonella* serovars from chickens in Brazil, volumes of imported chicken meat, and numbers of human infections in the importing country is necessary to assess previous indications of the risk to public health of imported *Salmonella* and AMR into the UK.

Here, we have addressed this evidence gap. In the present study we sequenced 183 *Salmonella* genomes from chickens in Brazil, covering a seven year span and from across Brazil, and 357 genomes from humans, domestic poultry, and imported Brazilian poultry products in the UK. These data were used to identify the most prevalent serovars in chickens from Brazil. We used these data along with 1,259 publicly available *Salmonella* genomes to investigate the evolutionary traits of *Salmonella* Heidelberg and *Salmonella* Minnesota, and assess their genetic relatedness with *Salmonella* from imported chicken meat and humans in the UK and elsewhere.

## Results and discussion

### Strain collection and *in silico S*. *enterica* serovar and MLST characterisation

**Fig 1:**
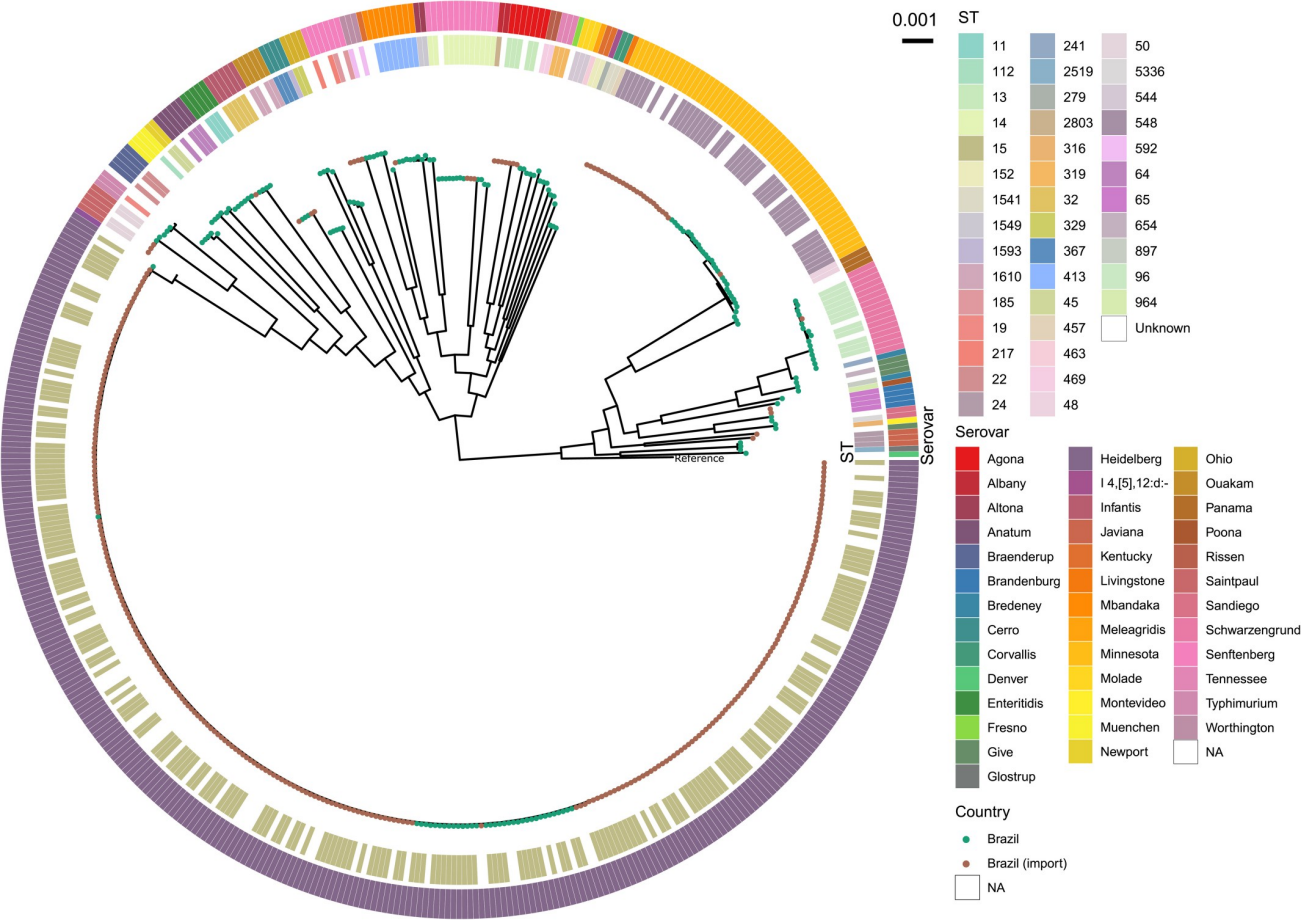
Genomic phylogeny of *S*. *enterica* isolates sampled in Brazil and imported chicken meat from Brazil. Maximum likelihood phylogeny of core genome alignment (253,248 informative sites) from 495 genomes of *S*. *enterica* isolated from Brazilian poultry production (tips—green) or known UK imports (tips—brown). Inner ring indicates sequence type (MLST) according to the key. Outer ring indicates serovar, predicted by SISTR. An interactive version of this figure with supporting metadata is available at https://microreact.org/project/brch-figure1.

A total of 183 *S*. *enterica* genomes, with varied genotypes and serovars, isolated in Brazil between 2012 and 2018 from thirteen states across the country were analysed. All isolates were obtained from broiler chicken samples sent to diagnostic laboratories for monitoring of *Salmonella* infection (see S1 Table). DNA from these isolates was subsequently extracted and sequenced using the Illumina NextSeq 500 platform with paired end 150bp reads. Through a combination of *de novo* genome assembly tools and *in silico* typing tools (see Methods), we determined that these isolates represented 36 different *S*. *enterica* serovars and 38 7-gene multi-locus sequence types (MLST). *Salmonella* Enteritidis (ST11; n = 4), which is the predominant cause of foodborne salmonellosis, accounted for only 2% of all isolates from Brazil. Similar to previous reports [4,5,10,11], *Salmonella* serovars Heidelberg (ST15) and Minnesota (ST548) were the most common with 37 and 28 genomes, respectively (S2 Table). Among the remaining isolates, serovars Schwarzengrund (ST96; n = 15), Senftenberg (multiple STs; n = 13), Mbandaka (ST413; n = 8), Anatum (ST64; n = 6), Braenderup (multiple STs; n = 5), Ouakam (ST1610; n = 5), Cerro (multiple STs; n = 4), Muenchen (ST112; n = 4) and Ohio (ST329; n = 4) were identified (S2 Table). Characterisation of all isolates, including the less frequent serovars (n<4) is available in Fig 1 and S2 Table.

To explore the link between poultry raised in Brazil and UK imported chicken, we also included 318 *Salmonella* genomes from poultry meat tested at the point of import into the UK from Brazil. These isolates were originally sequenced as part of imported food surveillance. Of the 318 genomes, 91% of the isolates were either *Salmonella* Heidelberg (n = 259) or *Salmonella* Minnesota (n = 26), whereas a single *S*. Enteritidis (ST11; n = 1) was detected. This high number of *S*. Heidelberg and *S*. Minnesota strains in poultry imported into the UK from Brazil is consistent with the results found in the newly sequenced genomes from poultry from Brazil and the previous reports cited above. Serovar and MLST prediction, and sequencing quality control metrics are available in S2 Table.

Both UK import and Brazilian poultry isolates are presented together in a maximum-likelihood phylogenetic tree (Fig 1). Genomes from import samples were closely related to samples from Brazil.

### Phylogenetic and temporal analysis of *Salmonella* Heidelberg and *Salmonella* Minnesota

Both *Salmonella* Heidelberg and *Salmonella* Minnesota serovars were the most common serovars in the 183 isolates sampled in Brazil and the 318 isolates obtained from imported Brazilian chicken in the UK, suggesting some selective advantage at the point of production (S1 Table; Country: “Brazil” & “Brazil (import)”). To explore this further, we constructed a representative dataset of global *Salmonella* Heidelberg and *Salmonella* Minnesota genomes. The *Salmonella* Heidelberg and *Salmonella* Minnesota datasets also included genomes from domestic UK poultry (30 and 9 respectively) to compare with imported Heidelberg and Minnesota (S1 Table; Source lab “APHA” [Animal and Plant Health Agency]). Additional genomes were selected to capture global genomic diversity through publicly available genomes on EnteroBase (see Methods and S3 Table).

**Fig 2:**
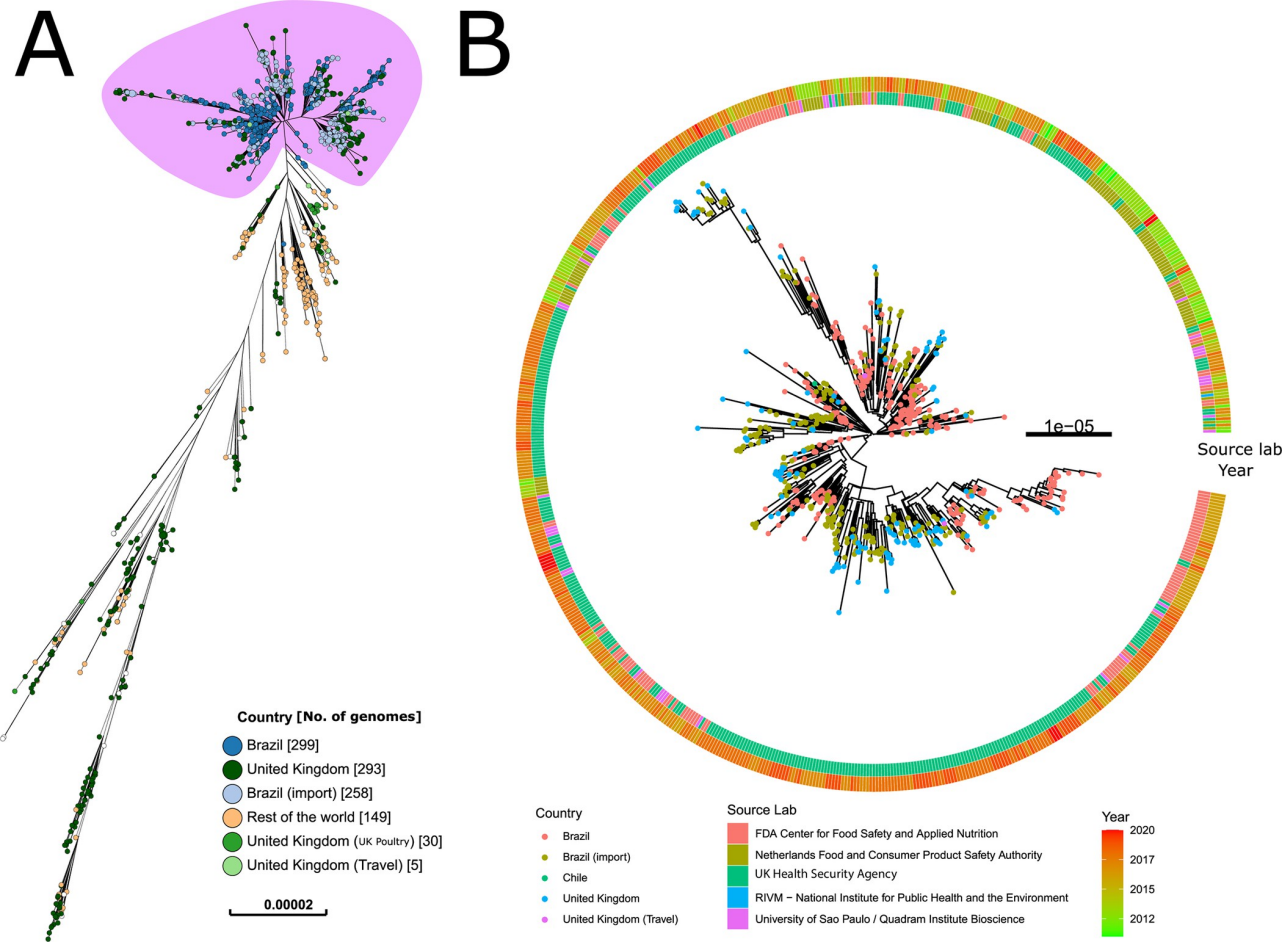
Phylogeny of global *Salmonella* Heidelberg including Brazil and UK (domestic/imported food/travel) isolates. (A) Maximum likelihood phylogenetic tree of *Salmonella* Heidelberg genomes, including isolates from Brazil, imported chicken meat from Brazil, domestic UK poultry, and representatives from the rest of the world. Nodes are coloured by country of origin as indicated in the key. An interactive version of this figure with supporting metadata is available at https://microreact.org/project/brch-fig2a (B) Maximum likelihood phylogenetic tree of genomes highlighted in lilac in part A. Tips were colour coded according to sample origin. Inner ring indicates the source lab. Outer ring indicates the collection year. An interactive version of this figure with supporting metadata is available at https://microreact.org/project/brch-fig2b.

By mapping sequenced reads against a *Salmonella* Heidelberg reference sequence CFSAN00324 (Accession GCA_000962725.1), we were able to construct a phylogenetic relationship between these genomes of different sources (Fig 2). All *Salmonella* Heidelberg genomes within this study that were isolated from Brazil, or known to be imported into the UK from Brazil via chicken meat, were within a single monophyletic group (Fig 2A). This clade (lilac) also included six domestic (UK) clinical isolates. All genomes from domestic UK poultry were outside the monophyletic group (Fig 2A; red). Notably, the genomes from Brazil were distant from those from the rest of the world. The phylogenetic trees of *Salmonella* Heidelberg, represented as transmission networks (S1A Fig) derived from Fig 2A and location data, confirm the clustering of Brazilian isolates. UK (domestic poultry) and other contextual isolates were separate from this central loop. The transmission network (S1B Fig) using the isolates’ source shows interplay between human (clinical), poultry and food but with no certain source.

The analysis was repeated for the *Salmonella* Minnesota genomes using a *Salmonella* Minnesota genome as a reference. The genomes from isolates that were collected from Portugal were sourced from a previous study assessing the presence of *S*. *enterica* in imported chicken products from Brazil [5]. These genomes were first identified through including all publicly available *Salmonella* Minnesota genomes listed on EnteroBase (S3 Table).

**Fig 3:**
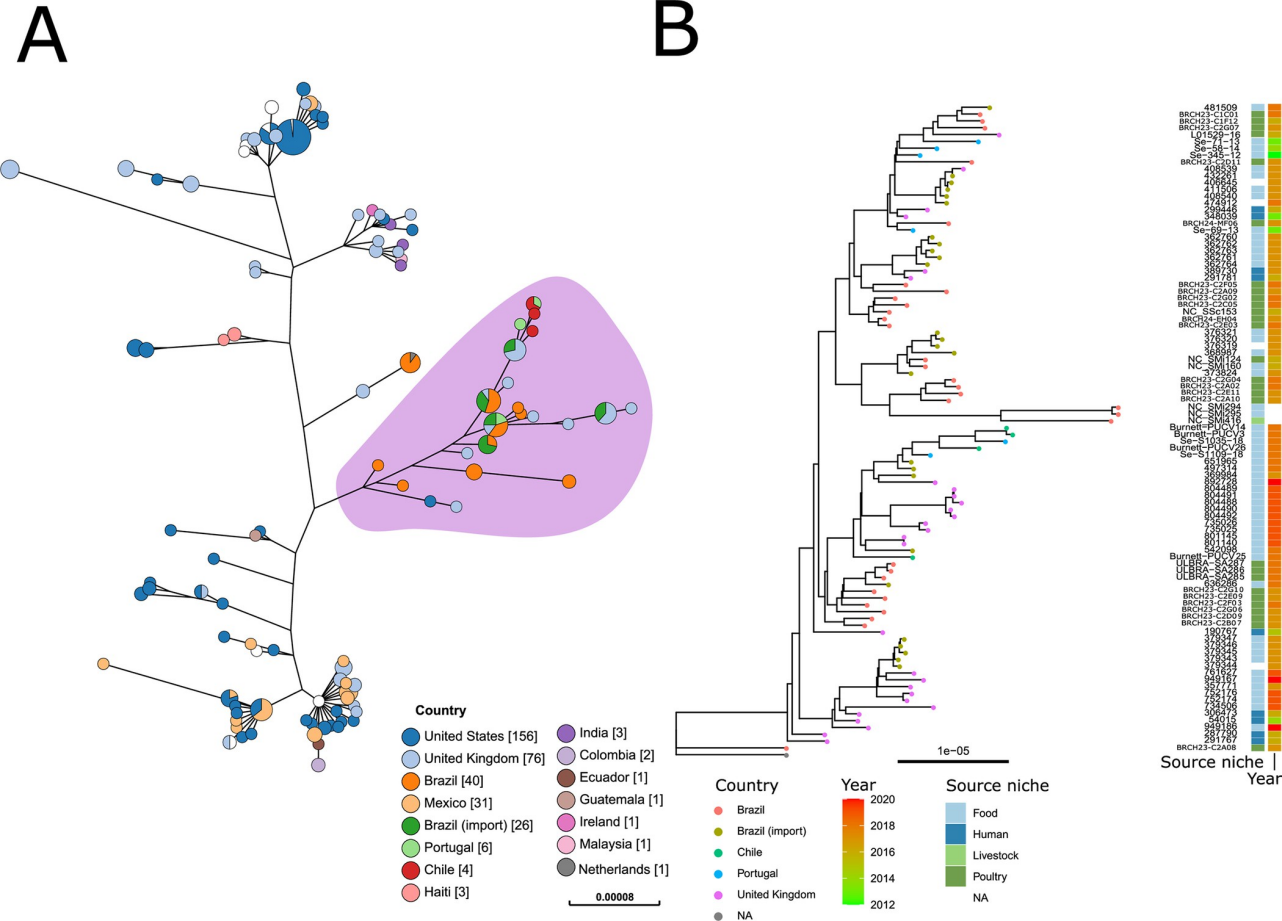
Phylogeny of *Salmonella* Minnesota. (A) Maximum likelihood phylogenetic tree of *Salmonella* Minnesota genomes, including isolates from Brazil, imported chicken meat from Brazil, and representatives from the rest of the world. Nodes are coloured by country of origin as indicated in the key. An interactive version of this figure with supporting metadata is available at https://microreact.org/project/brch-3a (B) Maximum likelihood phylogenetic tree of genomes highlighted in lilac in part A. Tips were colour coded according to sample origin. Inner ring indicates the source niche. Outer ring indicates the collection year. An interactive version of this figure with supporting metadata is available at https://microreact.org/project/brch3b.

Again, all genomes within this study that were obtained from Brazil, or known to be imported into the UK from Brazil via chicken meat, were within a single monophyletic group (Fig 3). This clade also included genomes from other imported poultry derived isolates (from Brazil to Portugal) and UK clinical isolates. Two *Salmonella* Minnesota genomes provided by the Animal and Plant Health Agency (APHA) were found in the monophyletic group (Fig 3A–lilac). Further investigation of the source of these isolates found that one (L01552-07) was a feed isolate from a UK feed mill, while the other (L01529-16) was part of an independent testing programme that did not sample UK chicken. The remaining seven *Salmonella* Minnesota sourced from APHA were found in two nodes elsewhere on the tree. Hence, as established with *Salmonella* Heidelberg, no *Salmonella* Minnesota directly attributed to UK domestic chicken was found within the monophyletic group.

The phylogenetic trees of *Salmonella* Minnesota, represented as transmission networks (S1C Fig) derived from Fig 3B and location data, illustrates the chain of transmission from Brazil, imported into the UK and Portugal. The transmission network (S1D Fig) using the isolates’ source shows interplay between human (clinical), poultry and food but with no certain source.

We also constructed separate time-dated phylogenies for the *Salmonella* Minnesota and *Salmonella* Heidelberg monophyletic groups to establish the possible date of emergence. Dating of the *Salmonella* Heidelberg monophyletic group was facilitated by additional older genomes (2010–2015) from imported poultry samples from Brazil into The Netherlands [4], suggested the *Salmonella* Heidelberg monophyletic group emerged between 1996 (CI95: 1968–2004) and 2004 (CI95: 1987–2006) (S2 and S3 Figs). The former date represents the date of the split from the closest known outgroup and the latter date represents the time to the most recent common ancestor (tMRCA) of all Brazil and UK imported isolates. This time frame is consistent with the tMRCA of the *Salmonella* Minnesota monophyletic group, which was calculated as 2006 (CI95: 1986–2007) (S4 and S5 Figs). Support for these dates can be seen in the root-to-tip charts and parameters illustrating the models’ convergence in S2 and S4 Figs. The effective sample size parameters for both models were appropriate (*Salmonella* Heidelberg: mu 244, sigma 288, alpha 244; *Salmonella* Minnesota: mu 394, sigma 501, alpha 274).

To determine the validity of these date ranges, a separate model was run for both monophyletic groups where isolation dates were fixed to a single date and in both cases a measure of the deviance information criterion reported that the randomised models were worse, indicating the temporal signal for both monophyletic groups is significant.

The time phylogenies’ results indicate that both monophyletic groups (Figs 2 and 3) represent recent expansions, likely aided by the introduction of the *Salmonella* Enteritidis vaccine into Brazil chicken production in 2003 [6]. The date for the *S*. Minnesota monophyletic group agrees with dates calculated previously, where the clade was designated “SM-PLII” and described as rapidly expanding in the beginning of the 2000s [24]. *S*. *enterica* control programmes and vaccines have been described as playing an important role in serovar shifts in poultry production since the 1930s [16].

### Human cases of *Salmonella* Heidelberg and *Salmonella* Minnesota in the UK

**Fig 4:**
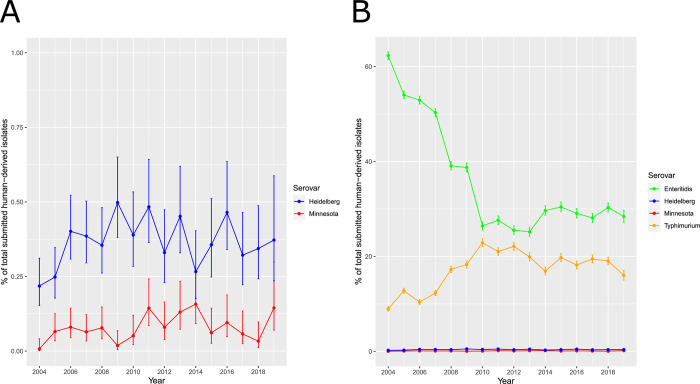
UKHSA *Salmonella* Heidelberg and *Salmonella* Minnesota trends in human salmonellosis cases in England and Wales. (A) Percentage over time of *Salmonella* Heidelberg and *Salmonella* Minnesota. (B) Percentage over time of four serovars causing salmonellosis in England and Wales.

Between 2004–2018, there were between approximately 8,100–14,000 confirmed reports of human *Salmonella* infections per year in England and Wales, reported by the UK Health Security Agency (UKHSA). Given the volume of chicken meat imported into the UK from Brazil, the contribution to this burden of disease from imported Brazilian chicken was investigated. In Brazil, in the present study and others [6,7], a decrease in *Salmonella* Enteritidis followed by an increase in *Salmonella* Minnesota and *Salmonella* Heidelberg serovars in poultry was observed after 2003, the year of introduction of the Enteritidis vaccine. No such rise for these serovars was observed in the historical data provided by UKHSA *Salmonella* surveillance of clinical cases in the UK (Fig 4), as would be expected if Brazilian imported chicken was a source of salmonellosis for humans in the UK. Indeed, there was a low and stable incidence of infections due to *Salmonella* Heidelberg and *Salmonella* Minnesota (≤0.5%) derived from humans between 2004 and 2019 (Fig 4A). This proportion is at least 20 times lower than infections due to *Salmonella* Enteritidis or *Salmonella* Typhimurium, which are the serovars accounting for the largest numbers of human cases (Fig 4B).

In addition, for *Salmonella* Heidelberg almost all of the UK-derived genomes which cluster with the Brazilian chicken sequences can either be attributed to cases with a recent history of foreign travel or are from imported chicken meat products (S3 Table); only six isolates were defined as clinical UK domestic samples. These findings indicate that *Salmonella* from imported Brazilian poultry products have not caused a major disease burden to UK consumers.

### Investigating AMR as a driver of the success of *Salmonella* Heidelberg and *Salmonella* Minnesota in Brazilian poultry

The predominance of *Salmonella* Minnesota and *Salmonella* Heidelberg, two unrelated *Salmonella* serovars, in Brazilian poultry could be driven by their common resistance to beta-lactams, sulfamethoxazole and oxytetracycline drugs. This was clearly observed at the genotypic level (S2 Table). To investigate if the same genetic elements were disseminating the resistance between the two serovars, long read sequencing of nine isolates was performed to resolve the genomic context of genes conferring AMR (S1 Table). These nine isolates (five Heidelberg and four Minnesota) were selected from within the UK collection (UKHSA) of isolates from raw chicken meat and other food products imported from Brazil and clinical samples, all of which clustered in the phylogenetic trees with the isolates from poultry tested in Brazil. All nine isolates carried *sul2* and *tetA* and seven carried *bla*_CMY-2_. Most of the genes were carried on IncC plasmids, including IncC plasmids carrying *sul2* and *tetA* (n = 4) and *sul2* and *tetA* in combination with *bla*_CMY-2_ (n = 4). *bla*_CMY-2_ was also found independently on IncI1 (n = 3) plasmids (S4 Table).

The genetic contexts of *sul2*, *tetA* and *bla*_CMY-2_ genes were investigated amongst IncC and IncI1 plasmids (S6A Fig). IncC plasmids had two gene configurations of *sul2* and *tetA* which were found in both *Salmonella* Heidelberg and *Salmonella* Minnesota isolates; in both configurations, the AMR genes were accompanied by a Tn3 transposase, as previously reported [25,26]. In one of the configurations, found only in *Salmonella* Minnesota isolates, an IS*91*-like insertion sequence (IS) was additionally integrated between *sul2* and *tetA*. In the second configuration, found only in *Salmonella* Heidelberg isolates, the *sul2/tetA/*Tn3 genetic block was in the reverse orientation to that in the first configuration (S6B Fig). In both IncC and IncI1 plasmids in both *Salmonella* Heidelberg and *Salmonella* Minnesota isolates, the *bla*_CMY-2_ gene was characterised by the upstream presence of IS*Ecp1* (S6C Fig), as previously reported [27,28], demonstrating the potential role of IS*Ecp1* in mobilising *bla*_CMY-2_ across serovars (S6C Fig).

Looking in the wider set of 183 Brazilian poultry genomes, a combination of *sul2*, *tetA* and *bla*_CMY-2_ was present in 77.8% and 75% of *Salmonella* Heidelberg and *Salmonella* Minnesota genomes, respectively, identified in 12 states. Only 2.5% of other *S*. *enterica* serovars in this dataset harboured *sul2* and *tetA* and none of them harboured *bla*_CMY-2_ (S2 Table).

To have a comparison for the gene and plasmid distribution in Brazil, a set of *Salmonella* genomes derived from poultry in Colombia was examined [29,30]. This demonstrated that Brazilian and Colombian *Salmonella* Heidelberg isolates shared a similar association of *sul2* and *tetA* with IncC and *bla*_CMY-2_ with IncI1 plasmids. However, only in Brazil was the association of *sul2*, *tetA* and *bla*_CMY-2_ with IncC plasmids observed, in both *Salmonella* Heidelberg and *Salmonella* Minnesota (S7 Fig).

**Fig 5:**
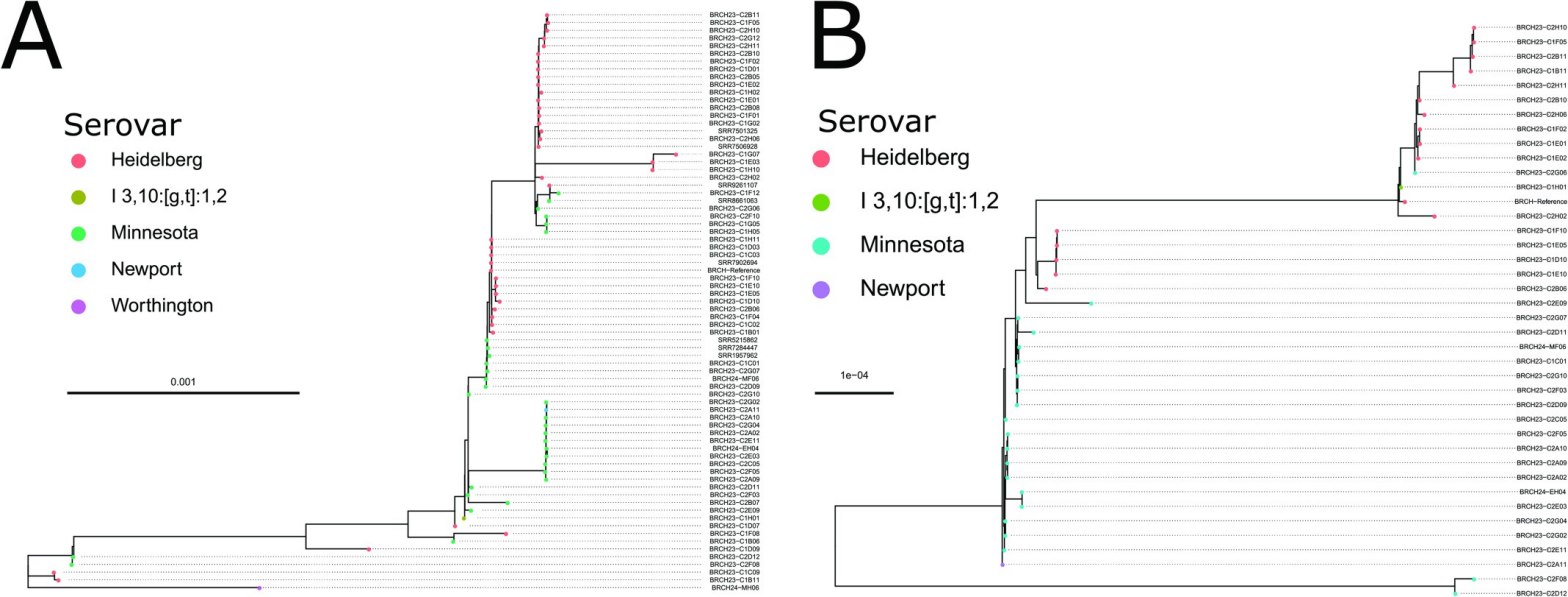
Phylogeny of *sul2*, *tetA* and/or *bla*_CMY-2_ carrying IncC plasmids in selected isolates. Selected isolates from imported Brazilian raw chicken and food products tested in the UK and chicken obtained in Brazil. (A) Maximum likelihood phylogenetic tree of 67 genomes identified with an IncC plasmid. Genomes were selected from Brazilian samples collected from poultry. Nodes are coloured by predicted serovar as indicated in the key. (B) Maximum likelihood phylogenetic tree of 39 genomes identified with an IncI1 plasmid. Genomes were selected from Brazilian samples collected from poultry. Nodes are coloured by predicted serovar as indicated in the key.

Phylogenetic analysis was performed for the IncC plasmids from Brazil and the UK. By mapping reads from Brazil isolates onto one IncC contiguous plasmid sequence recovered from the nine UK isolates, we found 67 out of 183 carry some version of the IncC plasmid, with clades largely specific with serovar (Fig 5A), suggesting the plasmids were introduced once into each serovar. Most Brazilian isolates were closely related to at least one of the nine isolates with long-read genome data but there were at least two clades that carry plasmid profiles not captured in the UK isolates. Similarly, a comparison for the IncI1 plasmid from Brazil and the UK showed 39 genomes out of 183 carried some version of the IncI1 plasmid, again with serovar specific clades. However, the genomes that harbour the IncI1 plasmid differ from those that harbour the IncC, and a different topology in the respective phylogenies was observed between the two types (Fig 5B).

We have so far described that the common element driving the prevalence of *Salmonella* Minnesota and *Salmonella* Heidelberg in Brazilian chicken production, both tested in the country of origin as well as the point of import into the UK, is the acquisition of AMR encoded by *sul2*, *tetA* and/or *bla*_CMY-2_. These genes are generally encoded on variants of IncC plasmid or were split between IncC and IncI1 plasmid types. This was observed in isolates collected across 12 states of Brazil, with the highest occurrence in those with the largest production of chickens in the country (Paraná, Santa Catarina, São Paulo and Mato Grosso do Sol). Comparisons of the genetic structure of these plasmids that have these general features show differences even within a small subset of the isolates described here (S6 Fig), and in comparing IncC plasmid sequences from this subset to all Brazil strains in the present study, there are likely to be more plasmid variants that are not fully described (Fig 5). We note that two isolates carrying these plasmids were from *Salmonella* serovars other than Minnesota and Heidelberg (i.e., Worthington and Newport) indicating that these plasmids can be found in other *Salmonella* serovars, although rare in this dataset. Laboratory work would be required to determine the relative rates of transfer of these plasmids into diverse *Salmonella* serovars. In addition, nucleotide BLAST searches (last accessed: 2 February 2021) using the above mentioned long read sequences (UKHSA) of *sul2* and *tetA* (n = 4), *sul2*, *tetA* and *bla*_CMY-2_ (n = 4) carrying IncC plasmids and *bla*_CMY-2_ carrying IncI1 plasmids (n = 3) were performed. The searches showed that for the *sul2* and *tetA* carrying IncC plasmids identified within the nine UKHSA long-read sequenced plasmids, similar plasmids (BLAST query cover ≥99% and identity ≥99%) were present in public genomes of *Salmonella* Heidelberg and *Salmonella* Typhimurium and in one publicly available *Salmonella* Minnesota genome. For *sul2*, *tetA* and *bla*_CMY-2_ carrying IncC, a similar plasmid to that observed in the UKHSA long-read genomes was found in three Minnesota isolates. For the two *bla*_CMY-2_ carrying IncI1 plasmids within the nine long-read UKHSA genomes, similar plasmids were present in *Salmonella* Heidelberg, *Salmonella* Typhimurium, *Salmonella* Kentucky, *Salmonella* Ohio, *Salmonella* Anatum, *Salmonella* Derby, *Salmonella* Brandenburg and some *Escherichia coli*. The publicly available genomes of *Salmonella* Heidelberg, *Salmonella* Typhimurium and *Salmonella* Minnesota that were similar to *sul2*, *tetA* and/or *bla*_CMY-2_ carrying IncC plasmids found within the nine UKHSA long-read genomes, originated from Brazil, USA and Russia. According to the metadata available, the genomes from Brazil and the USA originated from chicken related samples (S4C Table). The public genomes that were similar to the identified *bla*_CMY-2_ carrying IncI1 plasmids originated from a wide range of countries including Canada, Denmark, Japan, Uruguay and the USA. Similarly, there was a wide range of sources of isolation including poultry (predominantly), swine, bovine and human related samples (S4C Table).

In conclusion, as observed in other countries such as the United States, there have been important shifts in the *Salmonella* found in Brazilian poultry, where formerly *Salmonella* Enteritidis predominated. Here we suggest that the introduction of the *Salmonella* Enteritidis vaccine in poultry in Brazil led to serovar replacement, with *Salmonella* Minnesota and *Salmonella* Heidelberg becoming the dominant *Salmonella* in this setting. These two serovars are very different; *Salmonella* Heidelberg is a serogroup B *Salmonella* and *Salmonella* Minnesota, rarely observed outside of Brazilian poultry, is in serogroup L according to the Kauffman-White-Le Minor scheme [31]. Despite this difference, common carriage of the *bla*_CMY-2_, *sul2* and *tetA* genes, conferring resistance to beta-lactam, sulphonamide and tetracycline antimicrobials were detected, which were rarely seen in other *Salmonella* serovars co-circulating in Brazilian poultry production. What is not clear is why these very different serovars were able to acquire plasmids carrying these AMR genes and other serovars, co-circulating in Brazilian poultry and under the same selection pressures, were not; this suggests there are barriers to horizontal gene transfer of these plasmids. It is also possible that the selection for *Salmonella* Minnesota and *Salmonella* Heidelberg was aided by other genetic factors, such as other virulence genes.

*Salmonella* serovars Heidelberg and Minnesota were less associated with clinical disease in animals and presented low association with human disease in the data described here. Subsequent to the emergence of *Salmonella* Heidelberg and *Salmonella* Minnesota in Brazilian poultry, including that imported to the UK and Europe, human infection involving these serovars remains low in the UK. Unfortunately, there were no official data on *Salmonella* from human infections in Brazil to assess whether or not these serovars have an impact on Brazilian public health. Nevertheless, their mobilisable AMR features have potential broad dissemination capacity, biologically and geographically. Therefore, besides reduction of AMU, study of the barriers / factors / intermediate steps influencing the transfer of AMR genes can help in designing effective intervention strategies to reduce antimicrobial resistance. These insights were revealed through the concurrent evaluation of *Salmonella* genomes from Brazilian poultry, imported Brazilian chicken meat, and domestic human salmonellosis cases, and reinforce the importance of a One Health approach, which includes genomic surveillance.

## Methods

### Isolation and identification of *Salmonella*

A random selection of 183 deduplicated isolates from broiler faeces at the farm level was made in Brazil. These samples originated from diagnostic investigation of disease and monitoring of *Salmonella* shedding between 2012 to 2018 and included 13 Brazilian states. The sample set included longitudinal coverage from 2012–2018 for four major chicken-producing states (Paraná, Santa Catarina, São Paulo and Mato Grosso do Sol) and coverage from a larger number of states (n = 13) from 2017–2018. For *Salmonella* selective isolation, the faecal samples were inoculated in tetrathionate broth and incubated at 37°C for 24 h, and then plated on Xylose Lysine Tergitol 4 agar (XLT4, Difco) and CHROMagar *Salmonella* (Difco) (BD Diagnostics, Sparks, MD, USA) and incubated at 37°C for a further 24–48 h. The selected *S*. *enterica* colonies were identified by Matrix Associated Laser Desorption-Ionization–Time of Flight (MALDI-TOF) mass spectrometry and confirmed by lysine decarboxylase test. When available, 16 isolates per year from 2012–2018 and 10 isolates per state were randomly selected.

### Additional isolates of *Salmonella* and *Salmonella* genomes

The UK Health Security Agency (UKHSA) genomes originated from a nationwide programme of *Salmonella* surveillance in England and Wales [32]. From this programme, we included 318 *Salmonella* isolates from raw chicken meat and other food products from Brazil and samples at the point of import into the United Kingdom.

To provide context for *S*. Minnesota and *S*. Heidelberg, an additional 1,259 genomes were retrieved from EnteroBase (July 2020), see S3 Table. All known *Salmonella* Minnesota genomes available on EnteroBase at time of access (July 2020) are included in this study. These were selected from EnteroBase using the search terms “SISTR1 Serotype” equals “Minnesota” and “HC900 (cEBG)” equals “313”. To provide context for *S*. Heidelberg genomes sequenced in this study, we generated a subsample of all publicly available *S*. Heidelberg genomes (4,884—July 2020). These were selected from EnteroBase using the search terms “SISTR1 Serotype” equals “Heidelberg” and “HC900 (cEBG)” equals “536”. From all publicly available genomes, we selected one per cgMLST HierCC5 group, which equates to grouping all *Salmonella* Heidelberg genomes into distinct categories if they did not differ more than five cgMLST alleles and choosing one from each such group. HierCC cutoffs less than ten are described clustering epidemic outbreaks [33]. Within those groups, strains from the United Kingdom were preferred and then chosen based on the highest N50.

An additional 30 *Salmonella* Heidelberg isolates from chickens and 9 *Salmonella* Minnesota isolates from various sources were provided by APHA to provide the context of domestic UK poultry specifically.

Latin American genomes for genotype comparisons: Genomes of *Salmonella* Heidelberg (n = 20) and *Salmonella* Paratyphi B variant Java (n = 60) from previous studies in Colombia were collected from ENA Project Numbers PRJEB23610 and PRJEB31547 [29,30]. Colombia is a neighbouring country to Brazil with a similar *S*. *enterica* serovar distribution and resistance to sulphonamide, tetracycline and beta-lactam drugs.

To select UKHSA isolates for long read sequencing, those from the most prevalent serovars in Brazilian poultry were selected to examine the genomic context of AMR genes at a level of resolution not possible with short read data. A set of five representative isolates of *Salmonella* Heidelberg and four of *Salmonella* Minnesota was selected. The selection consisted of isolates collected in the UK and closely related to the genomes from Brazil. Details of UKHSA isolates are listed in S4 Table.

### Short read whole genome sequencing

Short read whole genome sequencing of isolates obtained from Brazilian chickens was performed as follows. Genomic DNA was purified with *DNeasy Blood & Tissue Kit* (Qiagen, Valencia, CA, USA) and used for 150 bp paired-end sequencing on the Illumina NextSeq 500 platform (Illumina, San Diego, CA, USA).

A modified Illumina Nextera low input tagmentation approach was used. 9 μl of TD Tagment DNA Buffer (Illumina Catalogue No. 15027866) was mixed with 0.09 μl TDE1, Tagment DNA Enzyme (Illumina Catalogue No. 15027865) and 4.01 μl PCR grade water in a master mix and 3 μl added to a chilled 96 well plate. Genomic DNA was normalised to 0.5ng/μl with EB (10mM Tris-HCl). 2 μl of normalised DNA (1ng total) was pipette mixed with the 5 μl of the tagmentation mix and heated to 55°C for 10 minutes in a PCR block.

A PCR master mix was made up using 4 μl kapa2G buffer, 0.4 μl dNTPs, 0.08 μl Polymerase and 4.52 μl PCR grade water, contained in the Kap2G Robust PCR kit (Sigma Catalogue No. KK5005, Sigma, St Louis, MI, USA) per sample and 9 μl added to each well need to be used in a 96-well plate. 2 μl of each P7 and P5 of Nextera XT Index Kit v2 index primers (Illumina Catalogue No. FC-131-2001 to 2004) were added to each well. Finally, the 7 μl of Tagmentation mix was added and mixed. The PCR was run with 72°C for 3 minutes, 95°C for 1 minute, 14 cycles of 95°C for 10s, 55°C for 20s and 72°C for 3 minutes.

Following the PCR reaction, the libraries were quantified using the Quant-iT dsDNA Assay Kit, high sensitivity kit (Catalogue No. 10164582) and run on a FLUOstar Optima plate reader. Libraries were pooled following quantification in equal quantities.

The final pool was double-SPRI size selected between 0.5 and 0.7X bead volumes using KAPA Pure Beads (Roche Catalogue No. 07983298001, Roche, Basel, Switzerland).

The final pool was quantified on a Qubit 3.0 instrument and run on a D5000 ScreenTape (Agilent Catalogue No. 5067–5588 & 5067–5589) using the Agilent Tapestation 4200 to calculate the final library pool molarity.

qPCR was carried out on an Applied Biosystems StepOne Plus machine (Applied Biosystems, Waltham, MA, USA). Samples to be quantified were diluted 1 in 10,000. A PCR master mix was made up using 10 μl KAPA SYBR FAST qPCR Master Mix (2X) (Sigma-Aldrich Catalogue No. KK4600), 0.4 μl ROX High, 0.4 μl 10 μM forward primer, 0.4 μl 10 μM reverse primer, 4 μl template DNA, 4.8 μl PCR grade water. PCR program run, 95°C for 3 minutes, 40 cycles of 95°C for 10s, 60°C for 30s. Standards were made from a 10 nM stock of Phix, dilution made in PCR grade water. The standard range was 20 pmol, 2 pmol, 0.2 pmol, 0.02 pmol, 0.002 pmol, 0.0002 pmol.

The pool was run at a final concentration of 1.5 pM on an Illumina Nextseq500 instrument using a Mid Output Flowcell (NSQ 500 Mid Output KT v2(300 CYS) Illumina Catalogue FC-404-2003) following the Illumina recommended denaturation and loading recommendations which included a 1% PhiX spike in (PhiX Control v3 Illumina Catalogue FC-110-3001). Data were uploaded to Basespace (www.basespace.illumina.com) where the raw data were converted to 8 FASTQ files for each sample.

### Genome assembly and annotation

Sequenced reads were screened using Kraken v2 [34] to confirm the species of each isolate as *Salmonella enterica*. De novo assembly of individual genomes was carried out using Shovill version 1.0.0 (https://github.com/tseemann/shovill, accessed November 2019), which internally corrected sequencing errors, performed genome assembly using SPAdes, and removed erroneous contigs. Assembly quality was assessed via QUAST v5.0.2 [35,36]. Draft genomes were annotated using Prokka v1.14 [37]. All raw sequence data from Brazilian isolates have been deposited in the European Nucleotide Archive (ENA) under BioProject accession PRJEB46151. All UKHSA sequence data are routinely deposited in the NCBI Sequence Read Archive (SRA) under BioProject accession PRJNA248792. All APHA sequence data are available in the ENA (accession PRJEB46896).

### Genotyping and characterisation of AMR genes and plasmid replicons

In silico Multi-Locus Sequence Typing was predicted using “MLST” v2.17.5 (https://github.com/tseemann/mlst, accessed November 2019) using the *Salmonella* MLST database hosted by EnteroBase [33]. Serovar prediction was performed with SISTR [38]. Antimicrobial resistance genes and plasmid replicon types were detected using ARIBA v2.14 [39] with default parameters. NCBI’s Bacterial Antimicrobial Resistance Reference Gene Database [40] and PlasmidFinder [41] databases were used for characterisation of AMR genes and plasmid replicons. Screening of point mutations conferring resistance to fluoroquinolones and polymyxins was made with staramr v0.7.1 (https://github.com/phac-nml/staramr).

### Phylogenetics of *Salmonella* Heidelberg and *Salmonella* Minnesota

The core genome alignment and single nucleotide polymorphisms (SNPs) were identified against the reference genome using Snippy v4.2.1 (https://github.com/tseemann/snippy, accessed November 2019) and visually inspected in Artemis 15 [42]. ClonalFrameML 1.12 [43] was used to define non-recombinant SNPs, which were used with IQTREE [44] to construct the final phylogeny. CFSAN00324 (Accession GCA_000962725.1) was used as the reference sequence in the analysis of *Salmonella* Heidelberg genomes. *Salmonella* Minnesota str. ATCC 49284 (accession no. CP019184.1) was used as the reference sequence in the analysis of *Salmonella* Minnesota genomes. Phylogenetic trees were visualised with ggtree v3.14 [45] and GrapeTree v1.7.0 [46].

### Time-dated phylogeny of *Salmonella* Heidelberg and *Salmonella* Minnesota

BactDating v1.0 was used to estimate the evolutionary rates and date the most recent common ancestor (tMRCA) [47] of Heidelberg and Minnesota isolates. Both root-to-tip plots and plots showing the convergence of the different parameters were generated through BactDating in R v3.6.1. Markov chain Monte Carlo chain lengths were run for 1 million cycles until effective sample sizes (ESS) over 100 were observed for mu, sigma, and alpha parameters, as suggested by the authors.

To determine that the estimated time of the most recent common ancestor (tMRCA) was driven by the data from the model itself, the analyses were repeated with dates for each genome all set to the same year. Both models were compared with deviance information criterion (DIC).

### Long-read sequencing

Nine isolates were selected from the UKHSA collection, five *Salmonella* Heidelberg and four *Salmonella* Minnesota for long-read sequencing using Oxford Nanopore Technology (ONT) MinION (ONT, Oxford, UK). Isolates were transported on Dorset egg slopes and cultured overnight on Luria Bertani broth. DNA was extracted using the FireMonkey High Molecular Weight DNA extraction kit (RevoluGen, Hadfield, UK) following manufacturer’s instructions. Length of DNA fragments was assessed using the Agilent 2200 TapeStation (Agilent, Santa Clara, CA, United States). DNA concentration was assessed with Qubit (Invitrogen, Waltham, MA, USA). Library preparation was made using the Rapid Barcoding kit (SQK-RBK004) from ONT (ONT, Oxford, UK) following manufacturer’s instructions. A total of 1020 ng of DNA per isolate was input into library preparation and 75μl of library loaded to an R9.4 MinION flow cell and sequenced for 72h. Long reads were base-called with Guppy v2.3.7 (https://nanoporetech.com/) and demultiplexed using Oxford Nanopore’s qcat command-line tool v1.1.0 (https://github.com/nanoporetech/qcat). Fastq reads were uploaded to Galaxy [48] for further analysis. Adapters were trimmed with Porechop v0.2.3 [49]. Quality of reads was calculated with Nanostat v0.1.0 and filtered with Nanofilt v0.1.0 (quality score >7, minimum read length >2000bp and headcrop 50nt) [50].

### Hybrid assembly of long-read sequenced strains

The short reads of the nine isolates selected from the UKHSA collection were available at NCBI’s SRA and were uploaded to Galaxy (Accession numbers in S1 and S4 Tables, BioProject: PRJNA248792). Short reads were previously obtained with the Nextera XT library preparation kit and the Illumina HiSeq 2500 platform. Unicycler v0.4.8.0 [51] was executed using bold, normal and conservative modes using the newly obtained long reads and previously available short reads. Circularisation of plasmid contigs was visually assessed with Bandage v. 0.8.1 [52].

Additionally, and to confirm the accuracy of Unicycler assemblies, short and long-read genomes were de novo assembled with Shovill v1.1.0 [53] and Flye v2.8 [54]. In all Unicycler and Flye assemblies, polishing was performed with Pilon v1.20.1 [55] over 10 iterations and Socru v2.2.2 [56] was used to identify assemblies with the least number of chromosomal mis-assemblies. Unicycler, Flye and Shovill assemblies were analysed for the presence of AMR genes and plasmid replicons with ABRicate v0.9.7 (github.com/tseemann/abricate), using the NCBI [40] and PlasmidFinder [41] databases. Among the Unicycler and Flye assemblies, those with a complete profile of AMR genes and plasmid replicons in comparison with Shovill assemblies were selected. Next, those with the least number of contigs and reduced length were selected and mapped against their respective short reads. Three metrics were obtained from BAM files: number of reads that mapped from end to start, indicating a circular sequence, number of reads with a correctly mapped mate, and reads with mates that did not map onto the assembled sequence. Genomes with the least number of the last metric were selected and further checked for completeness with CheckM v1.1.3. [57].

### Phylogenetics of *sul2*, *tetA* and/or *bla*_CMY-2_-carrying plasmids

Specific plasmid contigs from short read only genome assemblies were detected with MOB-Suite [58], and reads and contigs were mapped to representatives of IncC and IncI1 plasmids using Snippy v4.2.1 (https://github.com/tseemann/snippy, accessed November 2019). Representatives of IncC and IncI1 plasmids were selected from genome assemblies of long read sequencing (IncC: SRR7902694; IncI1: SRR7533524). The resulting core genome alignment was used with IQTREE v 2.0.3 [44] to construct the final phylogeny. Phylogenetic trees were visualised with ggtree [45].

### Genetic environment of *sul2*, *tetA* and *bla*_CMY-2_ in hybrid plasmid assemblies

The relatedness of complete hybrid plasmids sequence was assessed with Mashtree v0.57 [59]. The identification of the surrounding genes of *sul2*, *tetA* and *bla*_CMY-2_ was done with blastn v2.6.0 [60] and a custom database, which was built using plasmid annotations available at NCBI Nucleotide. Blastn was used for the comparison of the plasmid sequences. The similarities of plasmid sequences and annotations were plotted with genoPlotR v0.8.9 [61], and the mashtree plus the metadata were plotted using the ggtree v2.0.2 [45] package in R.

### Comparison of the prevalence of *S*. *enterica* serovars in Brazilian chicken and human cases of salmonellosis in the UK

Data of the incidence of *S*. *enterica* serovars in human cases in the UK were obtained from UKHSA *S*. *enterica* surveillance data. Visualisation of the incidence of *S*. *enterica* serovars in human cases in the UK were made with ggplot2 v3.3.2 [62] in RStudio v1.1.456.

### Comparison of AMR genes from two Latin American countries

The distribution of AMR genes *sul2*, *tetA* and *bla*_CMY2_ and the plasmid replicons IncI1 and IncC were compared between Brazilian and Colombian genomes using Base-R and ggplot2 v3.3.2 [62] in RStudio v1.1.456.

## Supporting information

S1 Table Contextual data for samples from Brazil obtained in the present study and those provided by UKHSA and APHA. (XLSX)

S2 Table Metrics of genomes from Brazil obtained in the present study and those provided by UKHSA and APHA. (XLSX)

S3 Table Collection of publicly available genomes of *Salmonella* Heidelberg and Minnesota collected from EnteroBase. (XLSX)

S4 Table Characteristics of UKHSA *Salmonella* Heidelberg and Minnesota strains selected for long-read sequencing. (XLSX)

S1 Fig Transmission networks base on phylogenetic trees. (PDF)

S2 Fig Temporal analysis *Salmonella* Heidelberg—root to tip and BactDating parameters. (PDF)

S3 Fig Temporal analysis *Salmonella* Heidelberg—timed phylogeny with confidence intervals. (PDF)

S4 Fig Temporal analysis *Salmonella* Minnesota—root to tip and BactDating parameters. (PDF)

S5 Fig Temporal analysis *Salmonella* Minnesota-—timed phylogeny with confidence intervals. (PDF)

S6 Fig Genetic environment of *sul2*, *tetA*, and/or *bla*_CMY-2_ in long read sequenced genomes. (A) Mashtree and comparisons of complete plasmid sequence harbouring *sul2*, *tetA* and/or *bla*_CMY-2_. On the left side is the Mashtree for eleven plasmids from nine isolates, note that there are two isolates with two plasmids each (indicated by [#1] and [#2]). Next to the tree are placed the serovars, plasmid replicons, target AMR, source of the bacterium isolation, and additional AMR. On the right side is the representation of the complete plasmid sequences, where homologous regions are indicated in dark red (% identity between 82% to 100%), and the genetic environment for *sul2* + *tetA* (orange rectangular box) and/or *bla*_CMY-2_ (green rectangular box). Genes are indicated by a square, with arrowheads showing the direction of transcription; AMR genes in red, insertion sequences in purple, hypothetical proteins in dark grey, other genes in light grey, replication start in cyan for IncI1, in blue for IncC plasmids. (B) Comparison of genetic environment for *sul2* + *tetA* in eight complete plasmids. There are two different gene configurations which are highlighted by the orange solid and dashed lines. The plasmid sequences were sorted by the serovars. (C) Comparison of genetic environment for *bla*_CMY-2_ in seven complete plasmids. Same gene configuration of *bla*_CMY-2_ is highlighted by the green line. The plasmid sequences were sorted according to the Mashtree order.
(PDF)

S7 Fig Comparison of *sul2*, *tetA*, and/or *bla*_CMY-2_ carriage in Brazil and Colombia. (PDF)
