# Supplementary figures and images for: Dynamics of Salmonella enterica and antimicrobial resistance in the Brazilian poultry industry and global impacts on public health
Source: PLoS Genet. 2022 Jun 2;18(6):e1010174. doi: 10.1371/journal.pgen.1010174 (PMC9162342; doi:10.1371/journal.pgen.1010174)

A

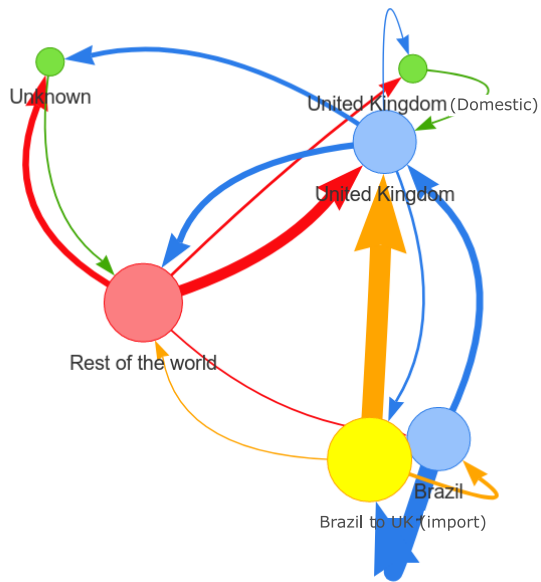

S. Heidelberg - Country

B

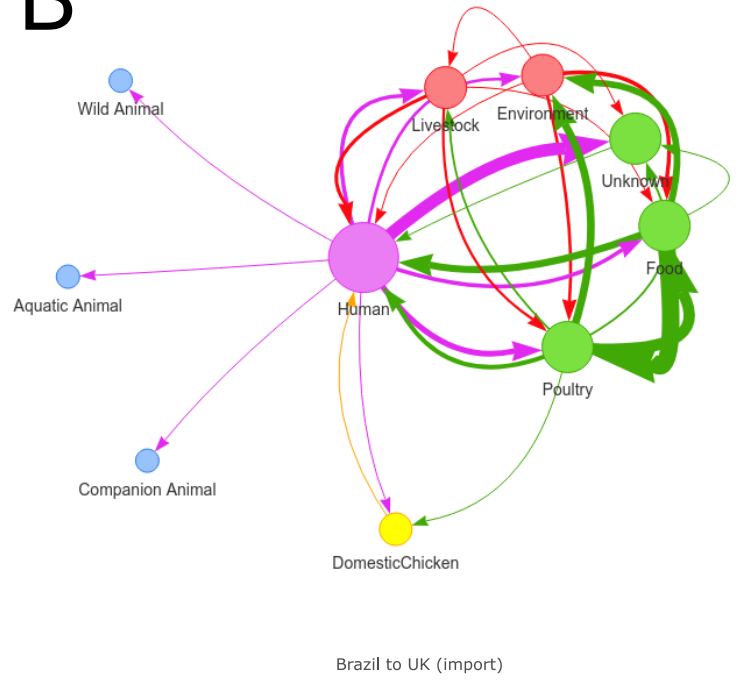

S. Heidelberg - Source

C

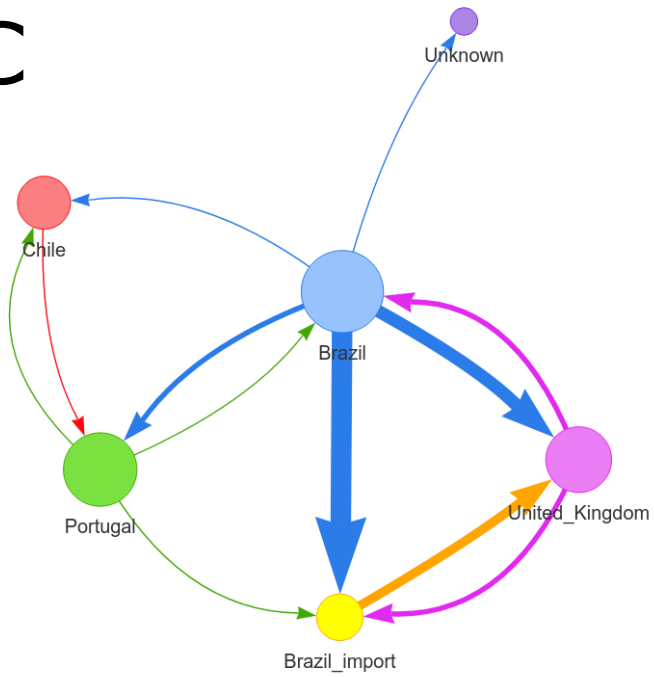

S. Minnesota - Country

D

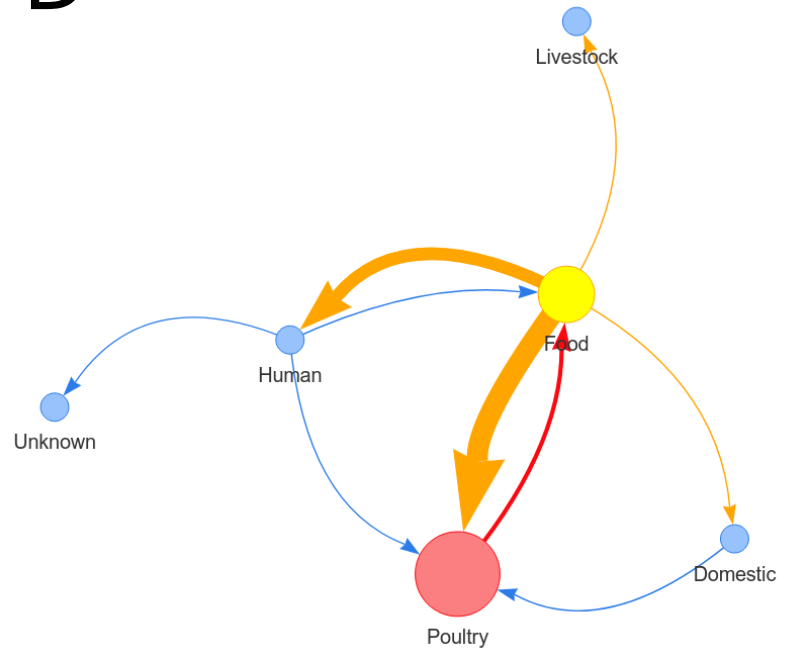

S. Minnesota - Source

Supplement: S1 Fig — (PDF) [file pgen.1010174.s005.pdf]

# A

Rate=1.61e-06,MRCA=2006.78,R2=0.31,p<1.00e-04

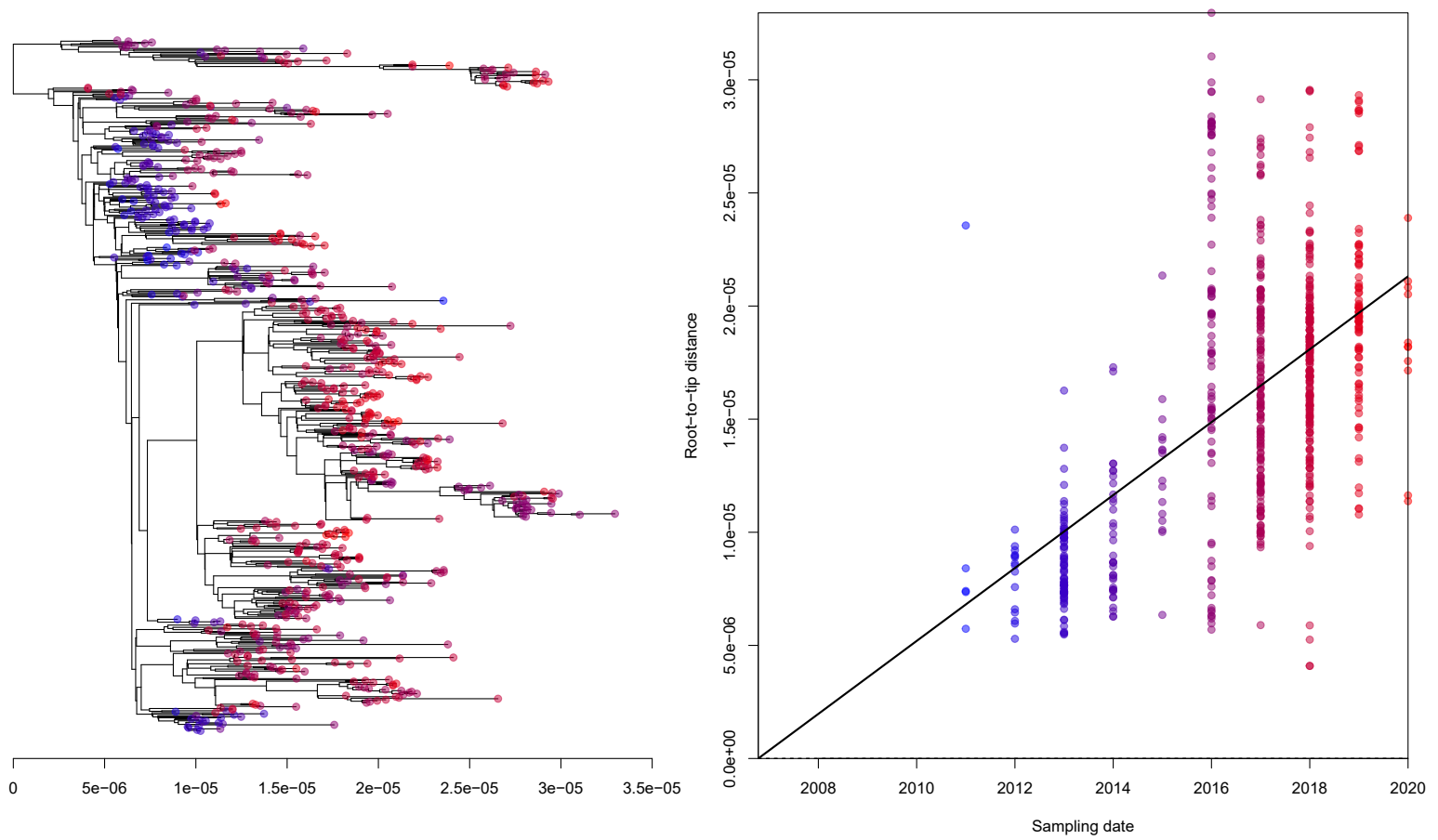

# B

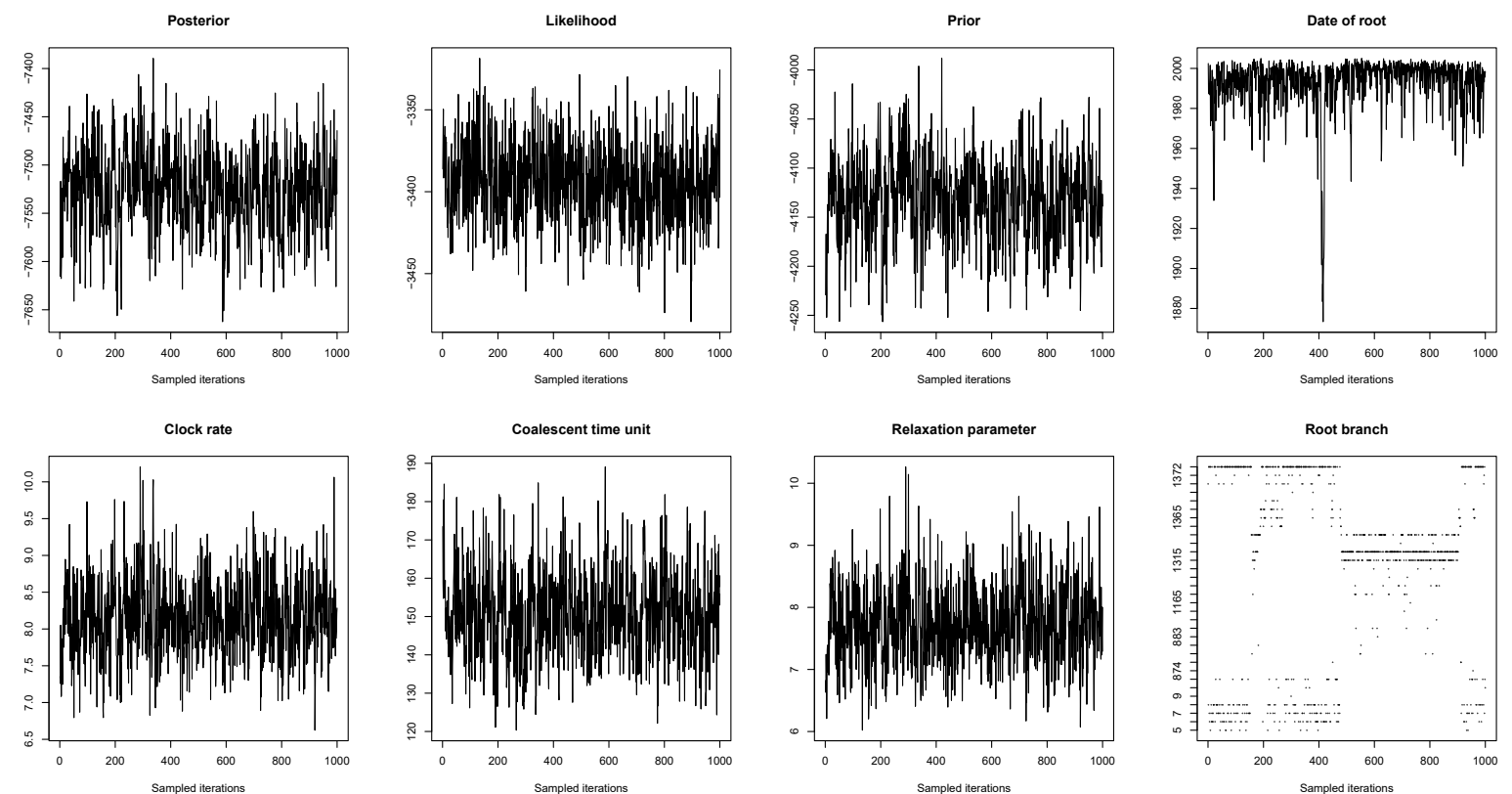

Supplement: S2 Fig — (PDF) [file pgen.1010174.s006.pdf]

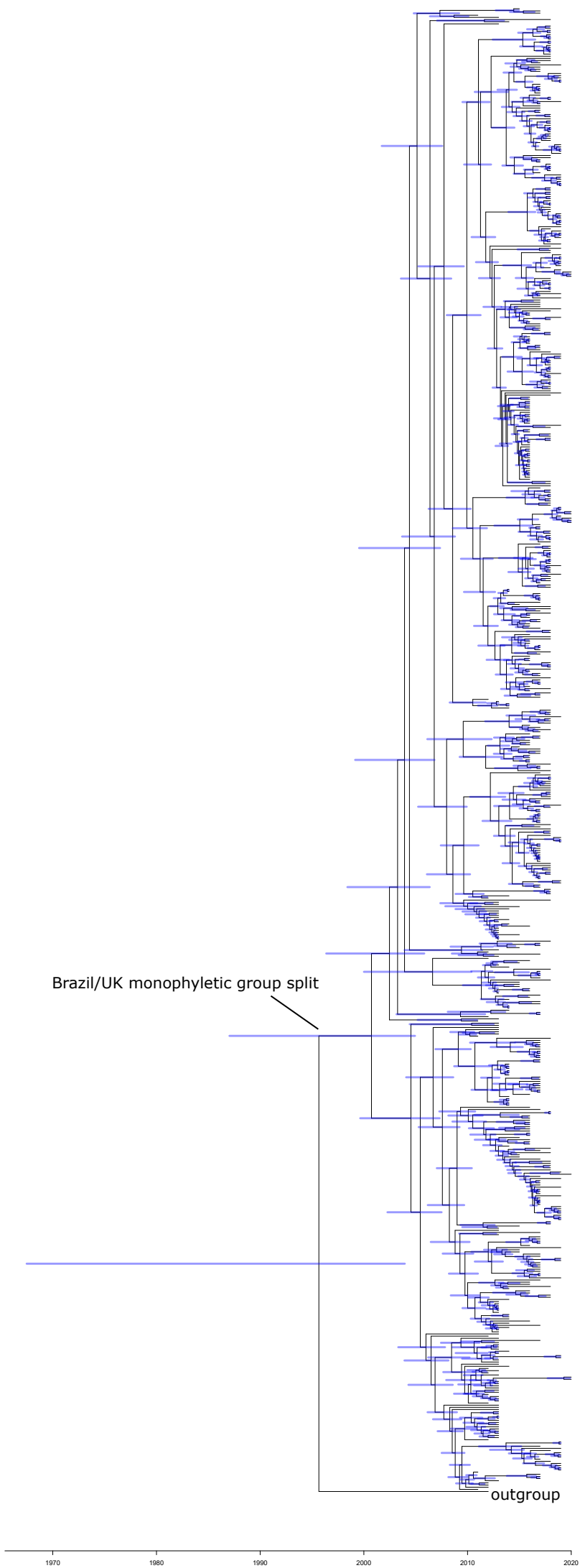

Supplement: S3 Fig — (PDF) [file pgen.1010174.s007.pdf]

A

Rate=1.08e+01,MRCA=2008.60,R2=0.27,p<1.00e-04

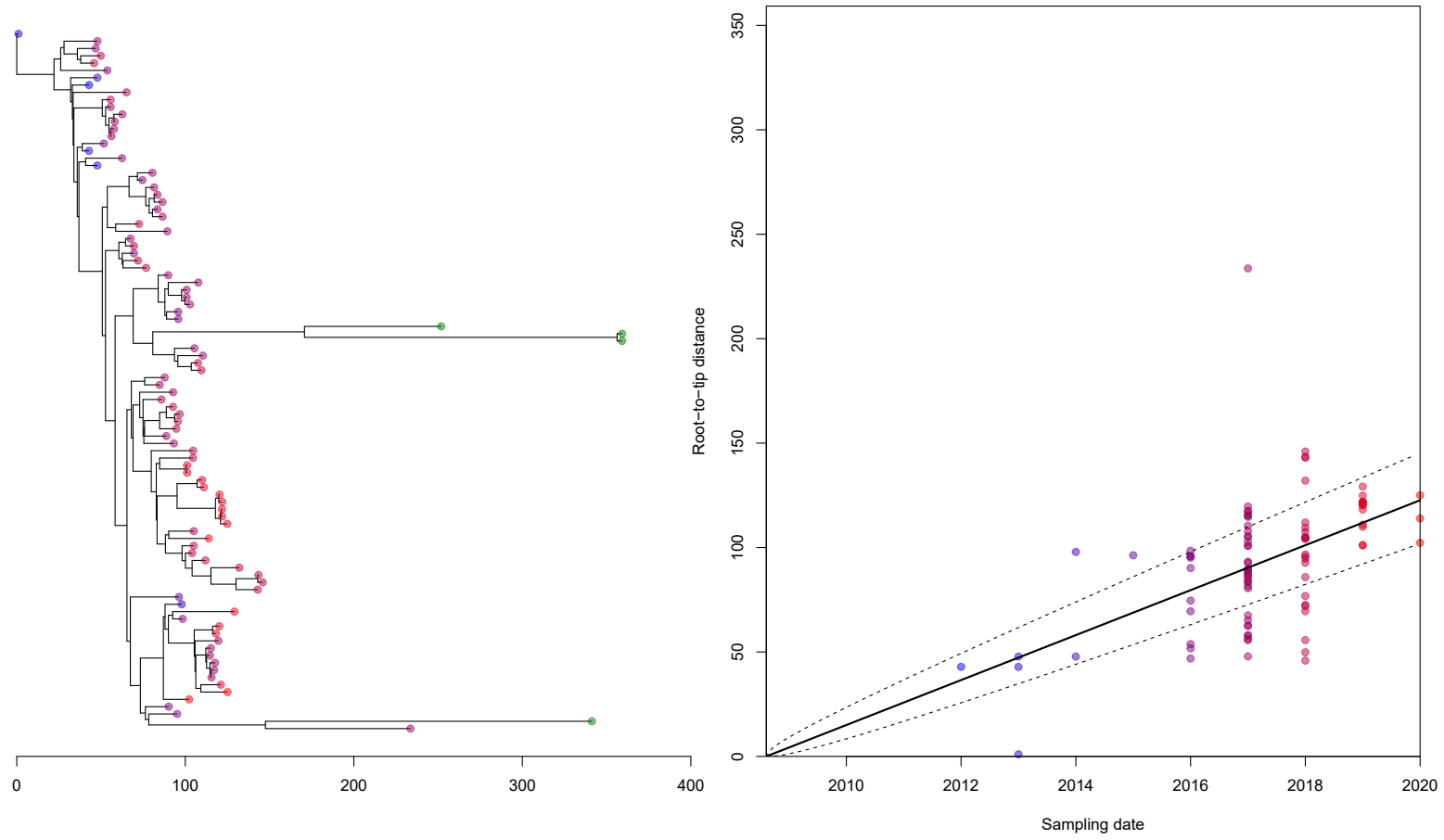

B

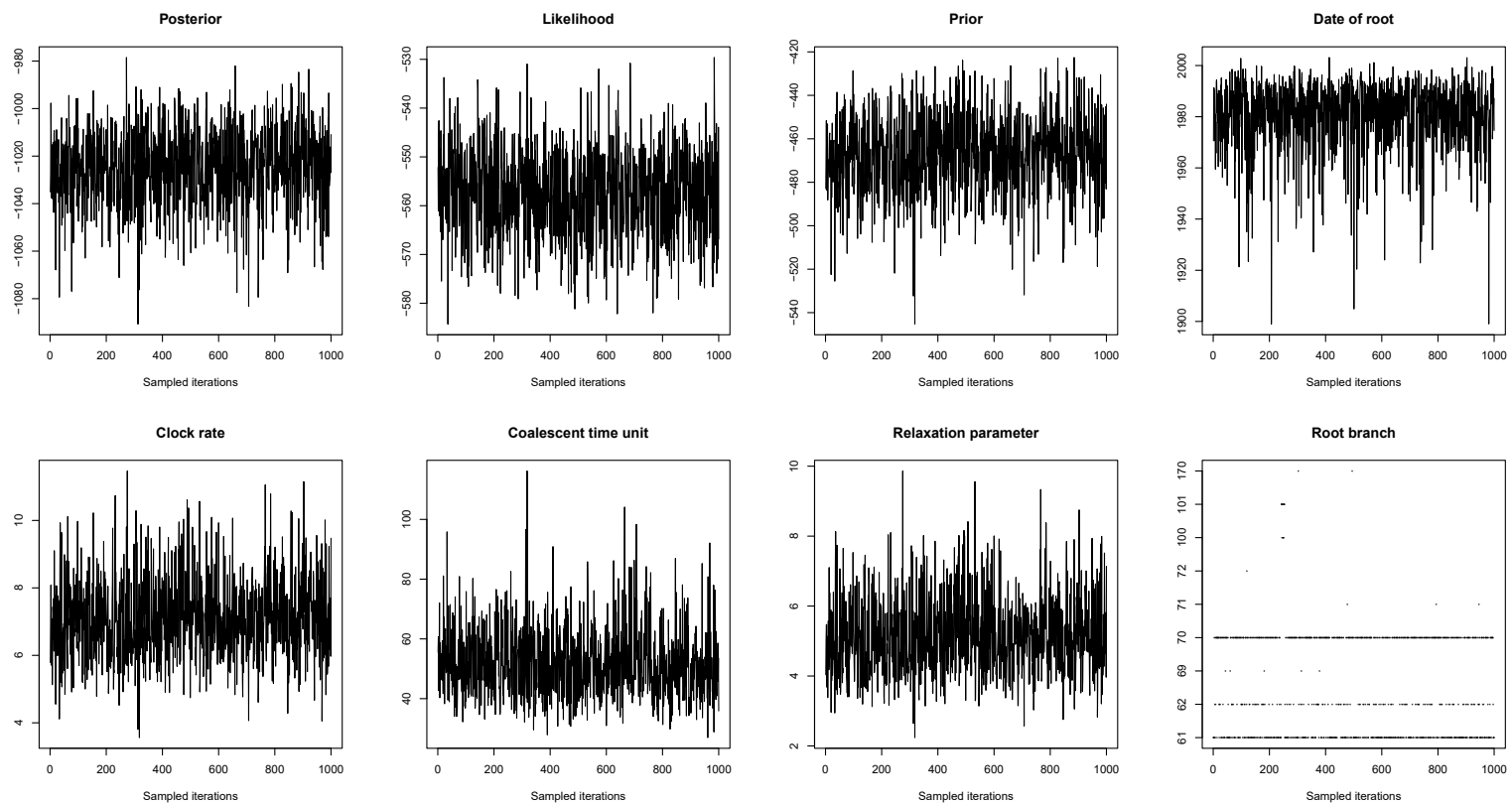

Supplement: S4 Fig — (PDF) [file pgen.1010174.s008.pdf]

Brazil/UK monophyletic group split

outgroup

1960 1970 1980 1990 2000 2010 2020

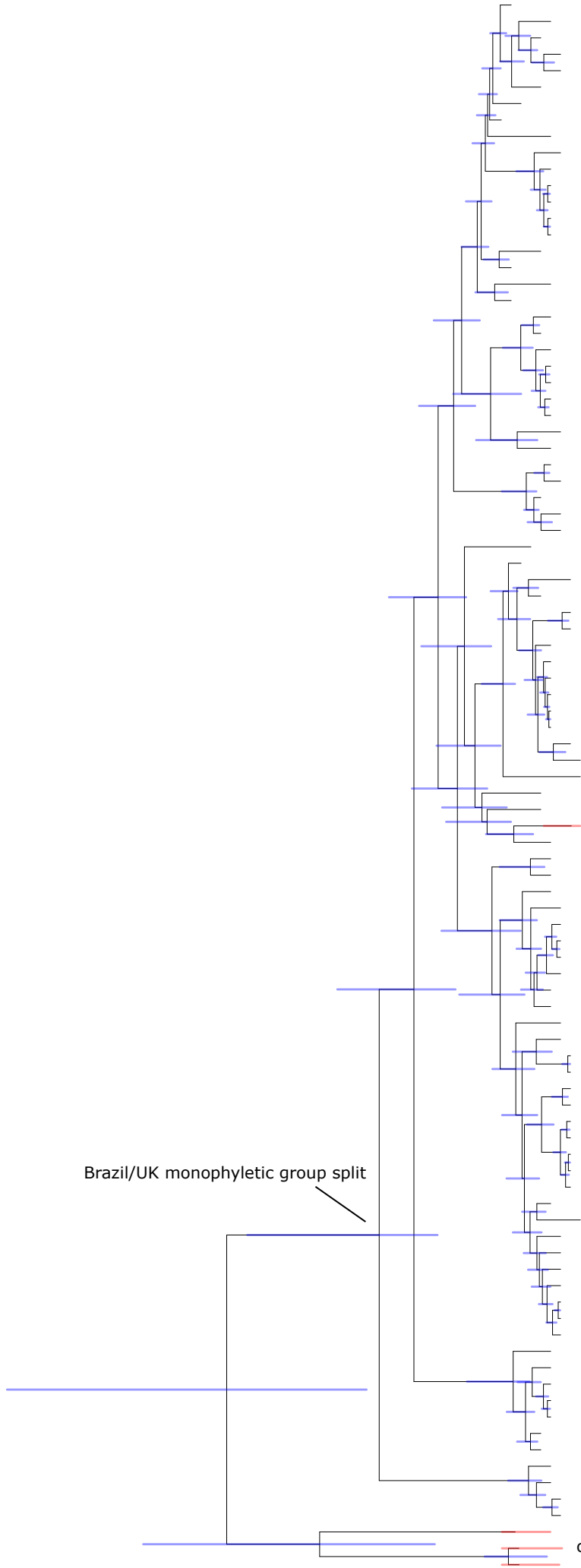

Supplement: S5 Fig — (PDF) [file pgen.1010174.s009.pdf]

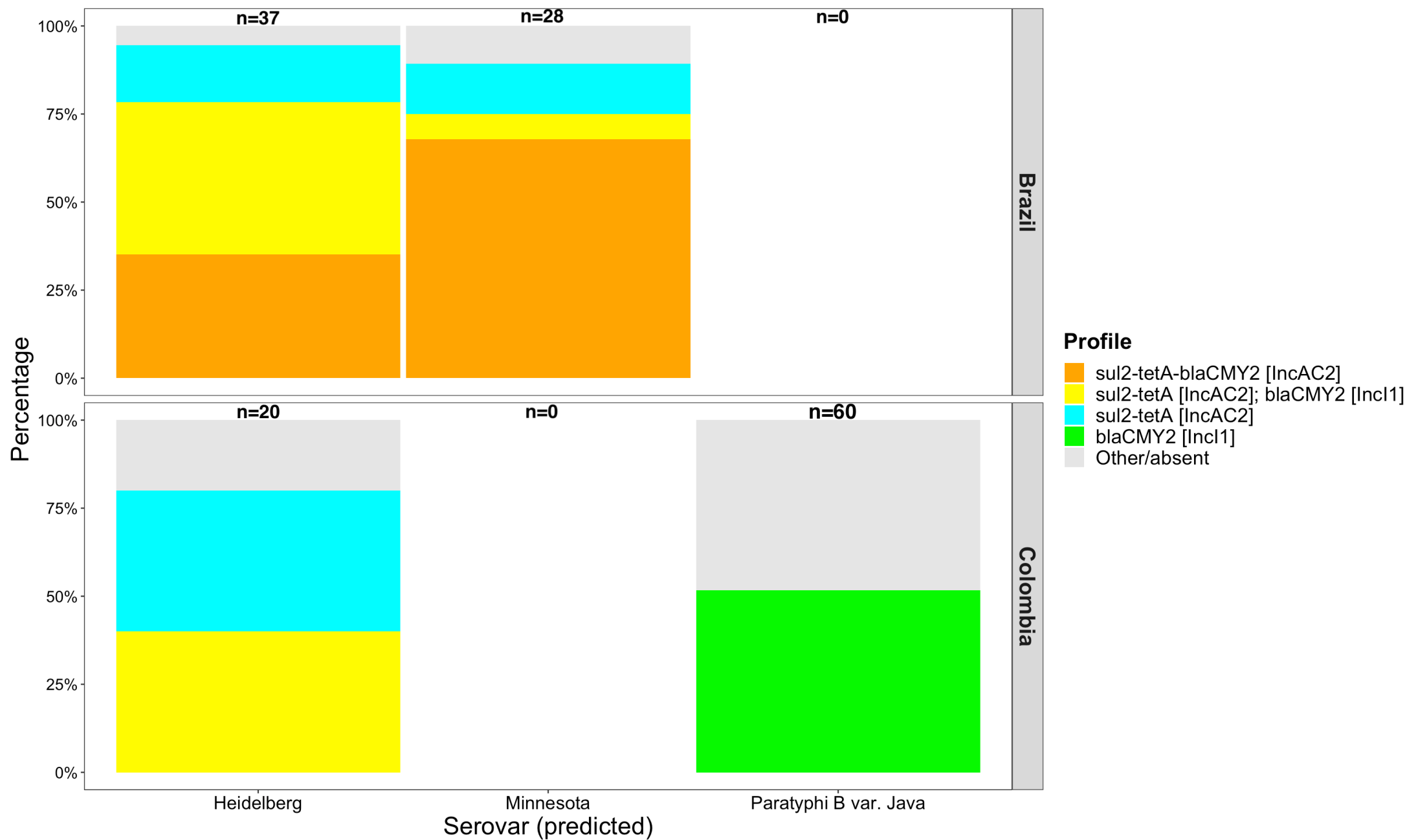

Supplement: S7 Fig — (PDF) [file pgen.1010174.s011.pdf]
