# Supplementary material for: Dynamics of Salmonella enterica and antimicrobial resistance in the Brazilian poultry industry and global impacts on public health
Source: PLoS Genet. 2022 Jun 2;18(6):e1010174. doi: 10.1371/journal.pgen.1010174 (PMC9162342; doi:10.1371/journal.pgen.1010174)
Supplement: S6 Fig — (A) Mashtree and comparisons of complete plasmid sequence harbouring sul2, tetA and/or blaCMY-2. On the left side is the Mashtree for eleven plasmids from nine isolates, note that there are two isolates with two plasmids each (indicated by [#1] and [#2]). Next to the tree are placed the serovars, plasmid replicons, target AMR, source of the bacterium isolation, and additional AMR. On the right side is the representation of the complete plasmid sequences, where homologous regions are indicated in dark red (% identity between 82% to 100%), and the genetic environment for sul2 + tetA (orange rectangular box) and/or blaCMY-2 (green rectangular box). Genes are indicated by a square, with arrowheads showing the direction of transcription; AMR genes in red, insertion sequences in purple, hypothetical proteins in dark grey, other genes in light grey, replication start in cyan for IncI1, in blue for IncC plasmids. (B) Comparison of genetic environment for sul2 + tetA in eight complete plasmids. There are two different gene configurations which are highlighted by the orange solid and dashed lines. The plasmid sequences were sorted by the serovars. (C) Comparison of genetic environment for blaCMY-2 in seven complete plasmids. Same gene configuration of blaCMY-2 is highlighted by the green line. The plasmid sequences were sorted according to the Mashtree order. (PDF) [file pgen.1010174.s010.pdf]

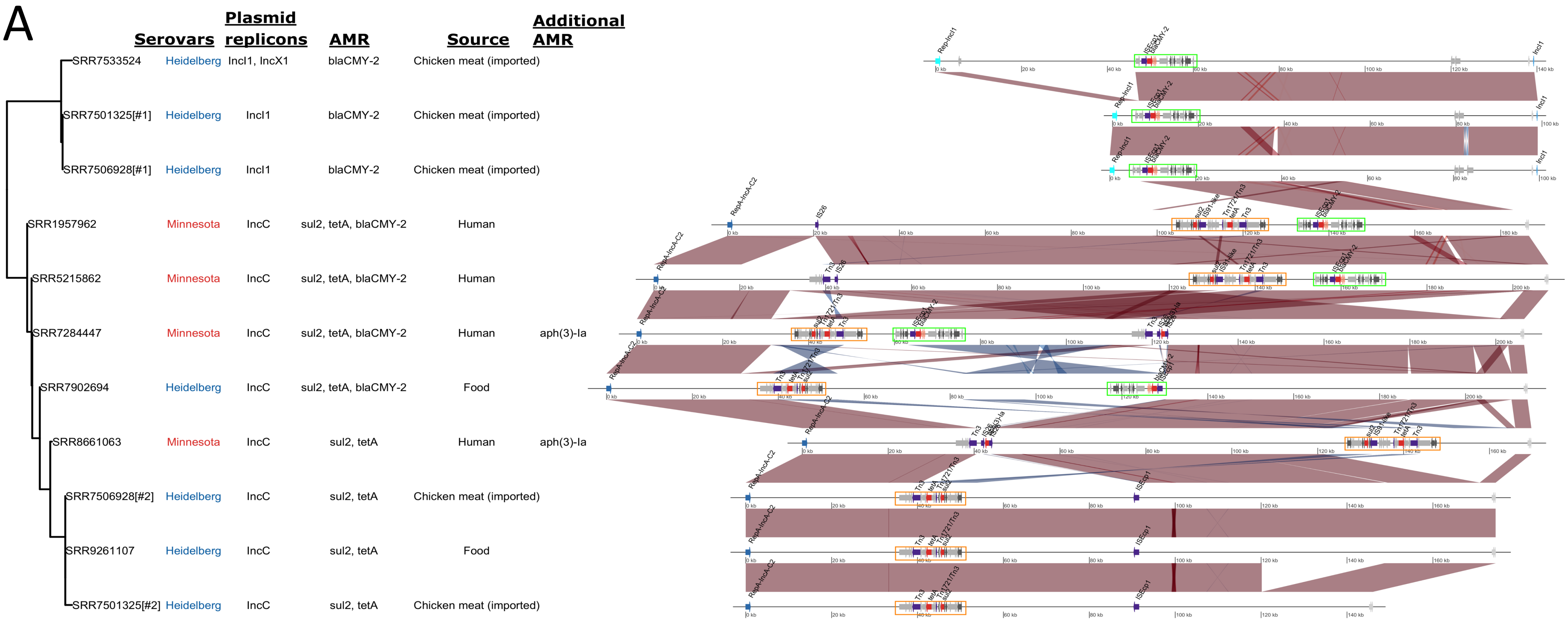
A
Serovars
Plasmid replicons
AMR
Source
Additional AMR
SRR7533524 Heidelberg IncI1, IncX1 blaCMY-2 Chicken meat (imported)
SRR7501325[#1] Heidelberg IncI1 blaCMY-2 Chicken meat (imported)
SRR7506928[#1] Heidelberg IncI1 blaCMY-2 Chicken meat (imported)
SRR1957962 Minnesota IncC sul2, tetA, blaCMY-2 Human
SRR5215862 Minnesota IncC sul2, tetA, blaCMY-2 Human
SRR7284447 Minnesota IncC sul2, tetA, blaCMY-2 Human aph(3)-Ia
SRR7902694 Heidelberg IncC sul2, tetA, blaCMY-2 Food
SRR8661063 Minnesota IncC sul2, tetA Human aph(3)-Ia
SRR7506928[#2] Heidelberg IncC sul2, tetA Chicken meat (imported)
SRR9261107 Heidelberg IncC sul2, tetA Food
SRR7501325[#2] Heidelberg IncC sul2, tetA Chicken meat (imported)

B

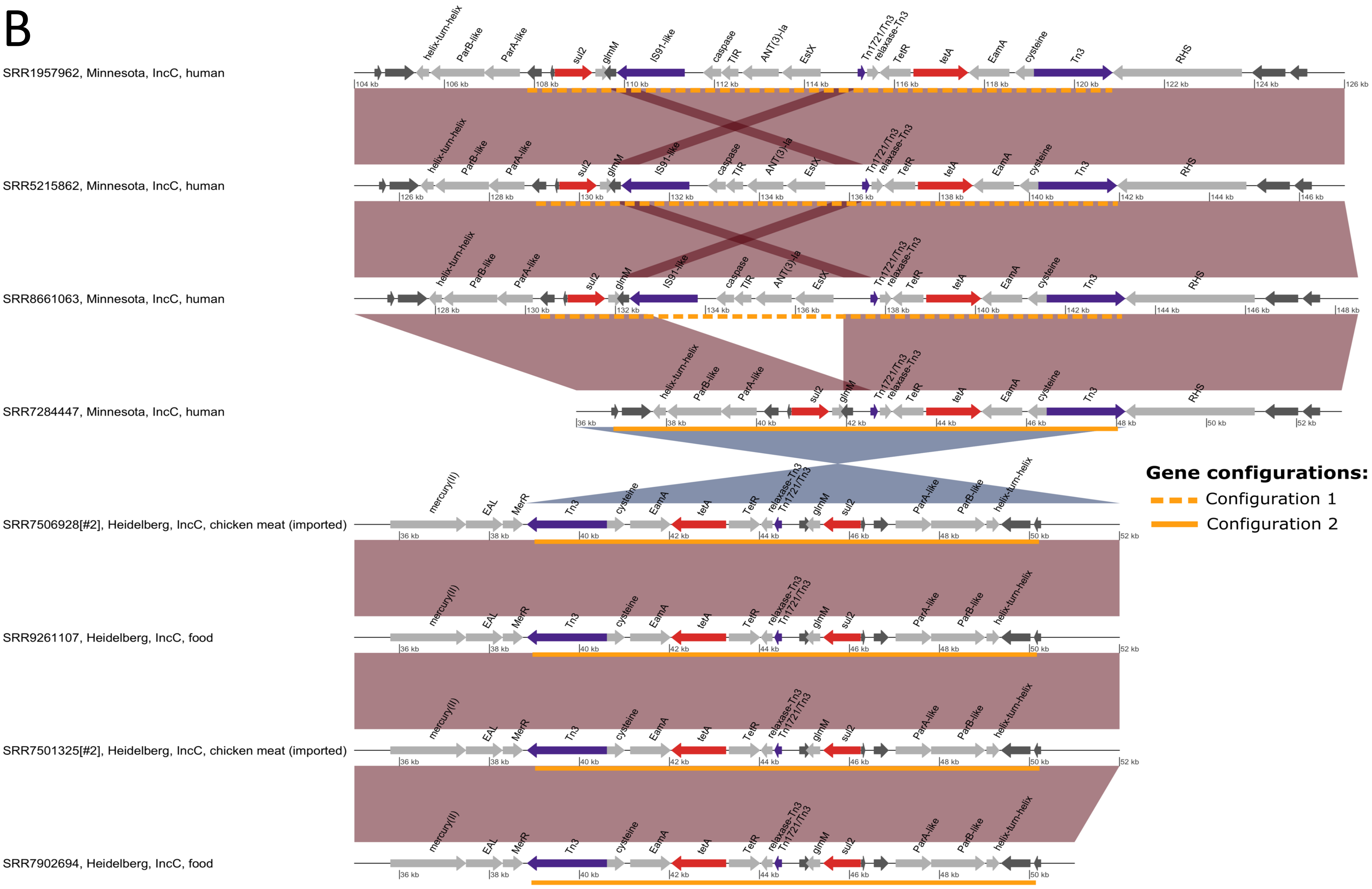
SRR1957962, Minnesota, IncC, human
helix-turn-helix
ParB-like
ParA-like
sul2
glmM
IS91-like
caspase
TIR
ANT(3)-Ia
EstX
Tn1721/Tn3
relaxase-Tn3
TetR
tetA
EamA
cysteine
Tn3
RHS
SRR5215862, Minnesota, IncC, human
SRR8661063, Minnesota, IncC, human
SRR7284447, Minnesota, IncC, human
SRR7506928[#2], Heidelberg, IncC, chicken meat (imported)
mercury(II)
EAL
MerR
SRR9261107, Heidelberg, IncC, food
SRR7501325[#2], Heidelberg, IncC, chicken meat (imported)
SRR7902694, Heidelberg, IncC, food
Gene configurations:
Configuration 1
Configuration 2

C

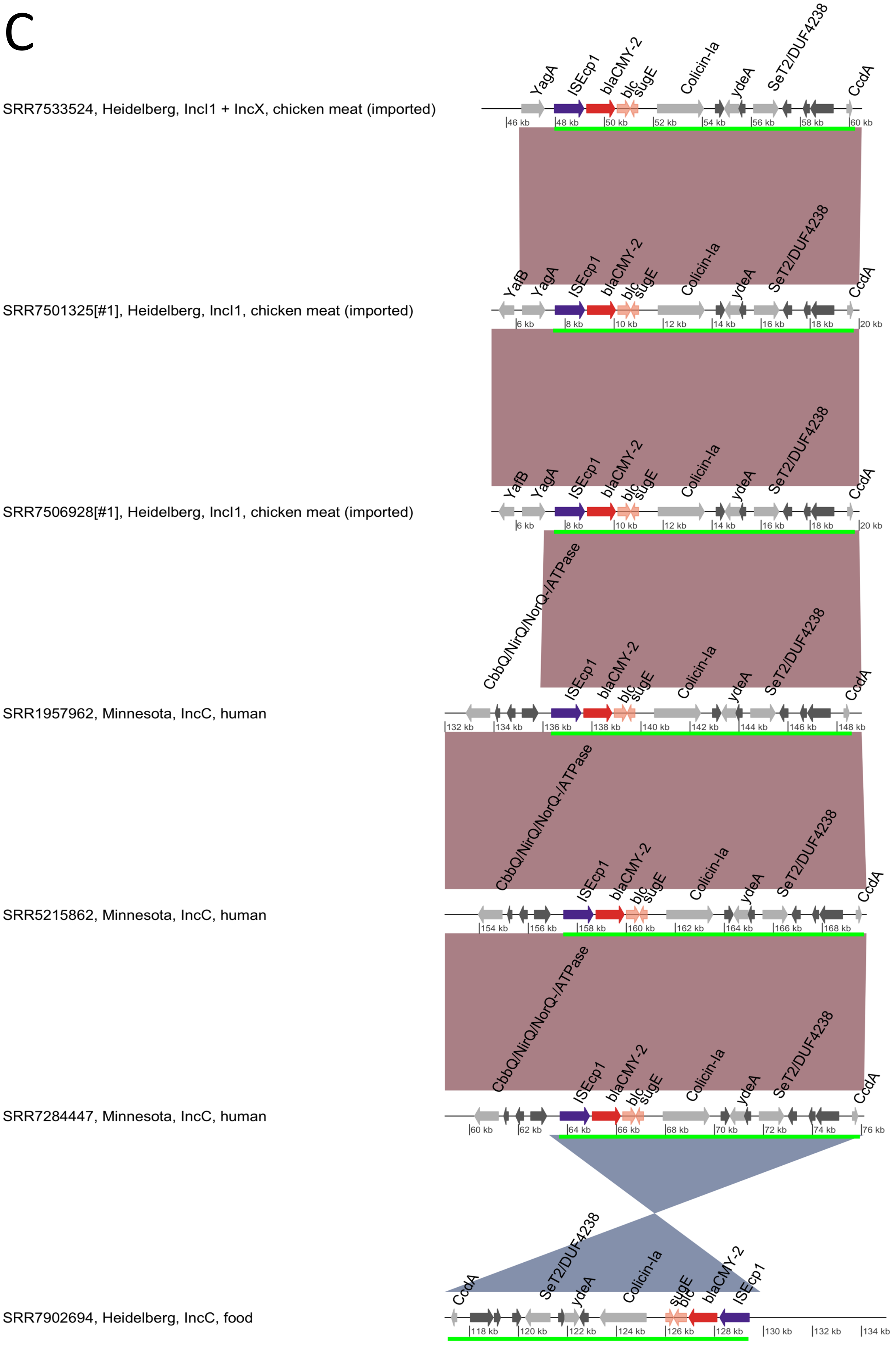

SRR7533524, Heidelberg, IncI1 + IncX, chicken meat (imported)
YagA
ISEcp1
blaCMY-2
blc
sugE
Colicin-Ia
ydeA
SeT2/DUF4238
CcdA
46 kb
48 kb
50 kb
52 kb
54 kb
56 kb
58 kb
60 kb
SRR7501325[#1], Heidelberg, IncI1, chicken meat (imported)
YafB
YagA
ISEcp1
blaCMY-2
blc
sugE
Colicin-Ia
ydeA
SeT2/DUF4238
CcdA
6 kb
8 kb
10 kb
12 kb
14 kb
16 kb
18 kb
20 kb
SRR7506928[#1], Heidelberg, IncI1, chicken meat (imported)
YafB
YagA
ISEcp1
blaCMY-2
blc
sugE
Colicin-Ia
ydeA
SeT2/DUF4238
CcdA
6 kb
8 kb
10 kb
12 kb
14 kb
16 kb
18 kb
20 kb
SRR1957962, Minnesota, IncC, human
CbbQ/NirQ/NorQ-/ATPase
ISEcp1
blaCMY-2
blc
sugE
Colicin-Ia
ydeA
SeT2/DUF4238
CcdA
132 kb
134 kb
136 kb
138 kb
140 kb
142 kb
144 kb
146 kb
148 kb
SRR5215862, Minnesota, IncC, human
CbbQ/NirQ/NorQ-/ATPase
ISEcp1
blaCMY-2
blc
sugE
Colicin-Ia
ydeA
SeT2/DUF4238
CcdA
154 kb
156 kb
158 kb
160 kb
162 kb
164 kb
166 kb
168 kb
SRR7284447, Minnesota, IncC, human
CbbQ/NirQ/NorQ-/ATPase
ISEcp1
blaCMY-2
blc
sugE
Colicin-Ia
ydeA
SeT2/DUF4238
CcdA
60 kb
62 kb
64 kb
66 kb
68 kb
70 kb
72 kb
74 kb
76 kb
SRR7902694, Heidelberg, IncC, food
CcdA
SeT2/DUF4238
ydeA
Colicin-Ia
sugE
blc
blaCMY-2
ISEcp1
118 kb
120 kb
122 kb
124 kb
126 kb
128 kb
130 kb
132 kb
134 kb
